# Red clover (*Trifolium pratense* L.) draft genome provides a platform for trait improvement

**DOI:** 10.1038/srep17394

**Published:** 2015-11-30

**Authors:** Jose J. De Vega, Sarah Ayling, Matthew Hegarty, Dave Kudrna, Jose L. Goicoechea, Åshild Ergon, Odd A. Rognli, Charlotte Jones, Martin Swain, Rene Geurts, Chunting Lang, Klaus F. X. Mayer, Stephan Rössner, Steven Yates, Kathleen J. Webb, Iain S. Donnison, Giles E. D. Oldroyd, Rod A. Wing, Mario Caccamo, Wayne Powell, Michael T. Abberton, Leif Skøt

**Affiliations:** 1 The Genome Analysis Centre, Norwich Research Park, Norwich, NR4 7UH, UK; 2 Institute of Biological, Environmental and Rural Sciences, Aberystwyth University, Gogerddan, Aberystwyth, Ceredigion SY23 3EB, UK; 3 Arizona Genomics Institute, School of Plant Sciences, University of Arizona, Tucson AZ 85721, USA; 4 Norwegian University of Life Sciences, Department of Plant Sciences, N-1432, Ås, Norway; 5 Laboratory of Molecular Biology, Department of Plant Science, Wageningen University, Droevendaalsesteeg 1, 6708PB Wageningen, The Netherlands; 6 MIPS/Institute for Bioinformatics and Systems Biology, Helmholtz Center Munich, Ingolstädter Landstrasse 1, Neuherberg, Germany; 7 Forage Crop Genetics, Institute of Agricultural Sciences, ETH Zurich, CH-8092, Zurich, Switzerland; 8 Department of Disease and Stress Biology, John Innes Centre, Norwich NR4 7UH, UK; 9 CGIAR Consortium Office 1000, Avenue Agropolis, F-34394, Montpellier, Cedex 5, France; 10 International Institute of Tropical Agriculture (IITA), PMB 5320, Oyo Road, Ibadan, Nigeria

## Abstract

Red clover (*Trifolium pratense* L.) is a globally significant forage legume in pastoral livestock farming systems. It is an attractive component of grassland farming, because of its high yield and protein content, nutritional value and ability to fix atmospheric nitrogen. Enhancing its role further in sustainable agriculture requires genetic improvement of persistency, disease resistance, and tolerance to grazing. To help address these challenges, we have assembled a chromosome-scale reference genome for red clover. We observed large blocks of conserved synteny with *Medicago truncatula* and estimated that the two species diverged ~23 million years ago. Among the 40,868 annotated genes, we identified gene clusters involved in biochemical pathways of importance for forage quality and livestock nutrition. Genotyping by sequencing of a synthetic population of 86 genotypes show that the number of markers required for genomics-based breeding approaches is tractable, making red clover a suitable candidate for association studies and genomic selection.

Red clover is one of the most important forage legume crops in temperate agriculture, and a key component of sustainable intensification of livestock farming systems. Its beneficial attributes in terms of high protein forage and reduced need for nitrogen fertilizer input can contribute to reduce the environmental footprint of grassland based agriculture^1^. Red clover provides good biomass yield for two or three seasons^2^. This limited persistency has been attributed to biotic and abiotic stresses^1^. There is thus an urgent need for improving our understanding of the genetic basis of these traits, as well as those affecting forage yield, quality and livestock nutrition, in order to facilitate genetic improvement.

In terms of available genomics resources, genome assemblies exist for the two model legumes, *Medicago truncatula* (Gaertn.) and *Lotus japonicus* L.^3^,^4^, and several legume pulse crops including common bean^5^, soybean^6^, chick pea^7^ and pigeon pea^8^. Genomics resource development in forage legumes are less advanced, but a transcriptome assemblies exist for example in alfalfa (*Medicago sativa* L.)^9^ and red clover^10^. For the latter a consensus genetic map, based primarily on SSR markers is also available^11^. Current evidence suggests that synteny between white clover and *M. truncatula* is more highly conserved than synteny between red clover and *M. truncatula*^12^, most likely due to the fact that the basic chromosome number of eight is conserved between *M. truncatula* and white clover, whilst red clover has seven.

Red clover is a diploid (2n = 2x = 14) species with a genome estimated to be 420 Mb^13^. It is heterozygous due to its gametophytic self-incompatibility system, and is difficult to inbreed without severe loss of viability and vigour^14^. A draft assembly of reads from 16 different genotypes was recently published^15^, consisting of 305 Mb in 267,382 scaffolds, and an N50 value of 2.4 Kb, using the same statistical criteria, as we have used here. While this assembly is a significant step forward, it is fragmented, and the scaffolds have not been anchored to a genetic or physical map. Here we report a chromosome-scale reference draft genome for a red clover genotype of the variety Milvus (Milvus B) by integration of Whole Genome Sequencing (WGS) of short-length reads, Sanger-based bacterial artificial chromosome (BAC) end sequences, a physical and two genetic maps. This assembly is the first within the major *Trifolium* genus, one of the largest in the Fabaceae family with 255 species^16^. After the model species *M. truncatula*, it is the second genome assembly of a forage legume at pseudo-molecule level. This will provide a great platform for advances in genomics studies of traits of biological and agronomic importance in forage crops.

## Results

### Red clover genome sequencing and assembly

The genotype Milvus B of red clover was the source of genomic DNA for the sequencing and construction of the physical map. WGS was assembled from paired-end and mate-pair libraries using the Platanus assembler^17^, which outperformed the results obtained by ABySS^18^ and SOAP2^19^. The number of contigs was lower and their contiguity statistics were higher in the assembly produced by Platanus. We also tested and discarded the option of using SOAP2 to scaffold the contigs produced by ABySS, and the use of a *Gap-Closer* as an additional final step. After discarding sequences shorter than 500 bp, ABySS assembled 376 Mb in 108K scaffolds, SOAP2 assembled 430 Mb in 102 K scaffolds, and ABySS contigs scaffolded with SOAP2 produced 452 Mb in 105 K scaffolds. Platanus assembled 309 Mb in 39,904 scaffolds. Half of the assembly was contained in 353 scaffolds (N50 = 223 Kb), while 1054 scaffolds longer than 50 Kbp contained another 25%, and a significant number of shorter scaffolds contained the remaining 25% of the genome (Supplementary Table 1, Supplementary Fig. 1). We observed that 87.1% of the Kmers in the ABySS + SOAP2 assembly were present in the Platanus assembly (Supplementary Table 2). We annotated 40,868 genes and 42,223 transcripts. Of those, 22,042 genes were anchored onto the seven chromosomes. Our final assembly with Platanus was smaller in total size than the others because the repeat content appeared fewer times, but without missing low copy regions, such as those rich in gene content. This is supported by the gene annotations and the K-mer spectras of reads for the different assemblies (Supplementary Fig. 2). In these figures, the areas under the Kmer spectra were coloured according to the number of times that such Kmers appeared in each assembly. Approximately 173, 74 and 41 Mb appeared once, twice and more than twice in the Platanus assembly, compared to approximately 206, 62 and 206 Mb in the ABySS+SOAP2. Also, we compared the annotation of the Platanus assembly with the annotation of the assembly by ABySS and SOAP2, which contained 66,250 proteins. Approximately 27% of these proteins were fully contained with a perfect match in other proteins, while the corresponding percentage was 2.8 in the Platanus annotation. The gene-space lengths were similar, with 138 and 148 Mb for the Platanus and ABySS assemblies, respectively. We aligned 93% of the proteins from the Platanus assembly to the ABySS + SOAP2 assembly. The proportion of aligned proteins was the same in the other direction. Finally, we aligned each proteome to the *M. truncatula* proteome and identified best-reciprocal-hits (BRH). Approximately 9% of the BRH with the Platanus proteome were not found among the BRH with the ABySS+SOAP2 proteome. The proportion of these exclusive BRH was identical in the other direction (i.e. 9%). Furthermore, the percentage of complete and partial core proteins reported by CEGMA^20^ was 85.48% and 95.56% for the Platanus assembly, and 78.2% of the previously published RNA-Seq reads^10^ mapped to this assembly. Finally, we compared our WGS assembly with an assembly previously published from a pool of 16 red clover varieties^15^. The composite nature of the original sample and the absence of genetic or physical maps limited the coverage of that assembly. The sample was sequenced to an average of 30×, and the assembly contained 267,382 scaffolds, of which 135,502 were longer than 500 bp, for a total of 268.2 Kbp including an important proportion of duplicated content (Supplementary Fig. 3).

We integrated our WGS with Sanger-based bacterial artificial chromosome (BAC) end sequences, and two genetic maps. The physical map contained 29,730 BACs, of which almost 23,000 were in contigs (77.3%). Singleton BACs amounted to 6,743. There were 2,440 contigs ranging in size from a few hundred kb to over 1.7 Mb. Originally, about 200 genetic markers from two maps^13^ (Supplementary Fig. 4) were anchored to the physical map. Here we aligned 1,031 of the 1,388 markers from the two maps to place 532 of the longest scaffolds totalling 153.4 Mb, and used the BAC-end sequences as markers to further link 330 unplaced scaffolds with already placed scaffolds from the same physical contig. After removing shorter sequences, the final version of the genome assembly consisted of 309 Mb including 164.2 Mb in 7 chromosome-length sequences or pseudo-molecules, plus 75.2 Mb in 542 scaffolds longer than 50 Kb. The seven pseudo-molecules ranged from 13.02 to 28.17 Mb (Supplementary Table 1).

### Genome annotation of the red clover genome

**Figure 1:**
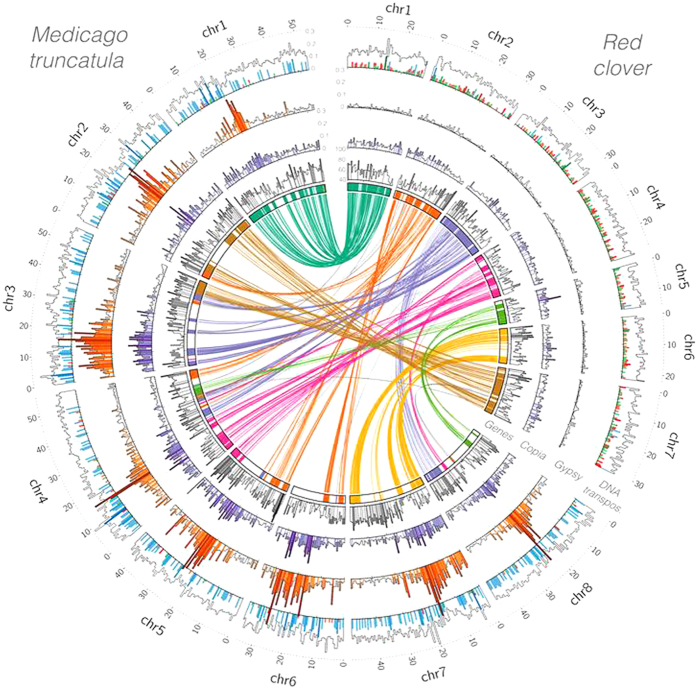
Structure of the red clover genome and synteny with the *M. truncatula* genome. (**a**) Lines connect duplicated genes between different chromosomes, and (**b**) concentric histograms of the density of genes (grey) and repetitive elements *Copia* (purple)*, Gypsy* (orange) and total DNA transposons (*hAT* in red, *TcMar* in green, and *MULE* in blue) in sliding windows of 1 Mb at 100 Kb intervals, only values in the top quartile are coloured.

We annotated 40,868 genes and 42,223 transcripts. Fig. 1 illustrates the spatial density of the 22,042 genes in the chromosomes (see Supplementary Fig. 5 for the individual chromosomes). A homologous protein in the UniprotKB database was found for 39,516 transcripts (93.6%), and 1,580 of the remaining transcripts (3.7%) had a novel ORF. A total of 31,576 transcripts (74.8%) was annotated with at least one GO term (Supplementary Table 3). The number of genes in red clover is lower than in *M. truncatula* (50,894) and soybean (56,044), but higher than in common bean (27,197). Red clover and *M. truncatula* have similar gene density, around 1.3 genes per 10 Kb (40,868 genes in 309 Mb and 50,894 genes in 389 Mb, respectively). However, the genes are not equally distributed in the genome. The mean value of the gene density in 10 Kb windows is similar in red clover, common bean and soybean (approximately 0.75 ± 0.96 genes per 10Kb), but lower than in *M. truncatula* (1.51 ± 1.13 genes per 10 Kb), because the latter has more intervals with many genes as observed in Fig. 1, and Supplementary Fig. 5. The CDS and exon lengths were similar in the four legumes, but the intron lengths were significantly longer in the legumes than in *Arabidopsis thaliana* (Supplementary Fig. 6).

### Comparative genomics in legumes

**Figure 2:**
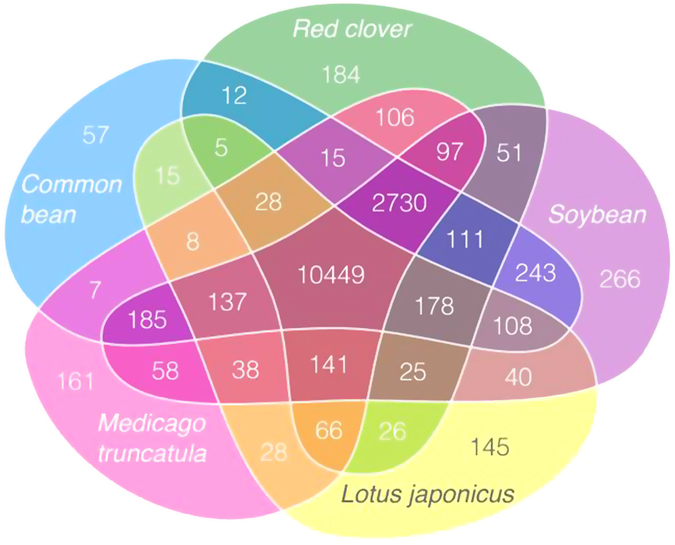
Venn diagram of gene clusters shared by the four Fabaceae species, red clover, *M. truncatula, L. japonicas*, soybean and common bean. The diagram was drafted with R/ggplot2^58^ using *facet_grid()*, with manual redrawing using the inkscape software (https://inkscape.org/).

**Figure 3:**
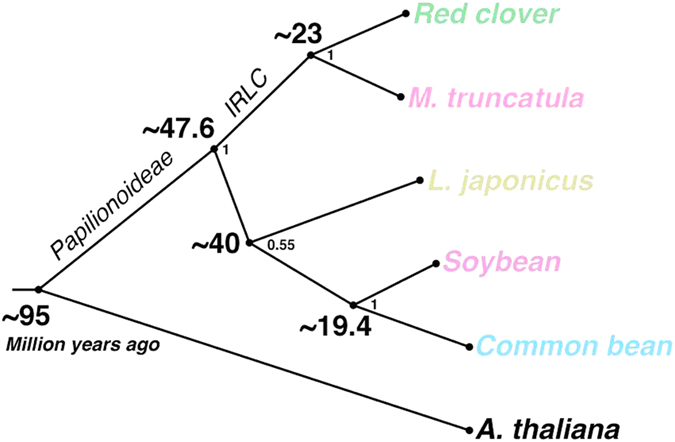
Divergence of red clover. Timeline in million years ago (MYA) for the divergence of red clover, *M. truncatula, L. japonicus*, soybean and common bean from each other, and from *Arabidopsis thaliana*.

There were 10,449 orthologous groups of genes common to red clover and four other Fabaceae species, and 2,730 groups common to all but *L. japonicus* (Fig. 2). The number of genes in each cluster was similar among these legumes, but differed from *A. thaliana*. Only 57 GO term clusters had more than twice the number of genes in red clover than in *M. truncatula*. This corresponded to 1,253 proteins mostly belonging to regulatory and transport families (Supplementary Fig. 7). We constructed a phylogenic tree on the basis of the alignment of the proteins of 818 single-copy clusters present in the five Fabaceae species and *A. thaliana*. We estimated that red clover and *M. truncatula* diverged around 23 million years ago (MYA) (Fig. 3), similar to that observed between common bean and soybean, which diverged ~19.4 MYA^5^. Furthermore, our analysis showed that the split of the papilionoid clades took place around 50 MYA, consistent with previous results^21^.

Macrosynteny was conserved between red clover and *M. truncatula*, which shared 17,278 gene pairs in 248 synteny blocks (Fig. 1). Red clover chromosomes 1 and 6 were almost entirely syntenic with *M. truncatula* chromosomes 1 and 7, respectively. The remaining five chromosomes had large synteny blocks each with two or three *M. truncatula* chromosomes (Fig. 1), as observed by others^12^. A synteny block was declared when there was at least 30 consecutive gene pairs. We quantified the divergence rates from gene pairs in the syntenic regions of each chromosome and observed a clear peak for the Kimura rates around 0.15 for the whole genome and equivalent divergence rates for each chromosome. We found 347 duplicated gene pairs in red clover (Supplementary Fig. 8), approximately three times less than the 963 gene pairs found in *M. truncatula*^3^. The pairs in red clover originated from duplication events around 12.7 MYA (Supplementary Fig. 9), thus after the divergence from *M. truncatula*, but we did not find a closer relation to repeat elements *Gypsy* or *Copia* in these duplicated gene pairs relative to all genes (Supplementary Fig 10).

The repeat content was 41.82% (Supplementary Table 4), which is slightly lower than previously reported^15^. We re-annotated the repeat elements in several legumes with recent versions of the databases and tools, and revised the fraction of repeats in *M. truncatula* to 48.7%, which is closer to the red clover values than the original annotation (30.5%)^3^. In common bean this value was adjusted to 72.1%, because we found a larger number of LINE and *Copia* transposable elements (TE) than in the original annotation (45.4%)^5^ and in soybean to 65.5%, similar to the values originally reported (61.5%)^6^. Class 1 TEs constituted 20.6% of the red clover genome (63.5 Mb). The fraction of LINEs and SINEs was similar in red clover and *M. truncatula*, but the proportion of *Gypsy* LTRs was much higher in *M. truncatula* (Fig. 1). Furthermore, the high *Gypsy* LTR regions coincided with high *Copia* LTR content. This striking difference is likely due to a recent burst of *Gypsy* activity, which took place 1–2 MYA in regions of *M. truncatula* not shared with red clover. This recent burst of *Gypsy* LTR activity, as well as a second one around 20 MYA that can be observed in both species, resulted in a much lower number of new *Gypsy* copies in red clover (Fig 1, Supplementary Fig. 9). Approximately 88% of the genes anchored in chromosomes had a *Copia* LTR within 10Kb, a third of them within 1 Kb. Approximately 39% of the genes had a *Gypsy* LTR within 10 Kb (Supplementary Fig. 10). Class 2 TEs constituted 19.1% of the red clover genome (58.9 Mb), which is similar to *M. truncatula*, but higher than in common bean and soybean (Supplementary Table 4). Fig. 1 highlights that although the total concentration of DNA transposons was similar, the distribution of families was not. Red clover had a higher proportion of *hAT*, *Stowaway* and *Pogo* transposons than any of the other species analysed, but in contrast to *M. truncatula* did not have *MULE* transposons.

### Gene clusters associated with forage nutrition traits

The high concentrations of isoflavones in red clover forage, particularly formononetin^1^, can have oestrogenic effects with adverse consequences for reproduction in ruminants, especially sheep^22^. Conversely, high formononetin content in red clover forage has been linked with higher live weight gains in lambs^23^. Formononetin concentration in red clover is under genetic and environmental control^24^. Four enzymes (Supplementary Fig. 11) are involved in formononetin biosynthesis, isoflavone-synthase (IFS1), 2-hydroxyisoflavanone dehydratase (HIDH), isoflavone-O-methyltransferase (IOMT), 2, 7, 4′-trihydroxyisoflavanone 4′-O-methyltransferase (HI4OMT), and two additional enzymes (Supplementary Fig. 11) are involved in the interconversion of formononetin conjugates isoflavone 7-O-glucosyltransferase (IF7GT) and isoflavonoid malonyl transferase (MAT7). Except for HIDH, which has had multiple copies since early in the evolution of plants and at least three recent duplication events in different loci of red clover (Supplementary Fig. 12), the genes encoding the other enzymes were distributed in five clusters in red clover and *M. truncatula*. Each cluster is dominated by one of the enzymes, and is surrounded by the same genes in both red clover and *M. truncatula* (Supplementary Fig. 13). Some genes encoding HI4OMT, IOMT and IF7GT were located in more than one cluster, but the genes in different clusters were distributed in different phylogenetic branches, and appear to encode distinct groups of isoenzymes and to have evolved independently prior to the Fabaceae divergence (Supplementary Figs 14–18). Five IOMT genes were clustered on *M. truncatula* chromosome 5, and red clover scaffold 1068 (3 genes) and scaffold 29975 (2 genes). The IOMT cluster includes two copies of *tRNA pseudouridine synthase* (TruA) in both species (Supplementary Fig 13). Five HI4OMT genes clustered in *M. truncatula* chromosome 4 embedded among several genes with unknown function. The five homologous genes in red clover were in different unplaced scaffolds, which may form a cluster too. The IFS1 cluster is on red clover chromosome 3 and *M. truncatula* chromosome 4, and is formed by four IFS genes, as well as two IOMT genes and two HI4OMT genes from phylogenetic branches different to the genes in the previous clusters. An *Auxin-response 3* transcription factor and a *cellulose synthase A* gene are also located in the IFS1 cluster in both species. The IF7GT cluster contains three contiguous IF7GT genes on red clover chromosome 2 and *M. truncatula* chromosome 5. IF7GT genes have expanded in soybean and common bean, but not in other analysed legumes. Finally, the MAT7 cluster on red clover chromosome 6 is formed by five contiguous MAT7 genes plus a sixth one 200 Kb upstream. The latter is contiguous to an expansin gene, a duplicated F-box transcription factor, and three IF7GT genes from a different phylogenetic branch than the previous IF7GT genes. The *M. truncatula* MAT7 cluster on chromosome 7 has an equivalent structure except that two IOMT genes are located between the MAT7 genes, physically linking both pathways. There are three homologous genes in red clover in unplaced scaffolds. Furthermore, there are eight additional IOMT genes distributed in eight unplaced scaffolds in red clover, though they belong to the same phylogenetic branch (Supplementary Fig. 15). Remarkably, this branch contains 18 genes in soybean, only four in common bean, and four pairs in *M. truncatula* chromosomes 1 and 7, including the described pairs in the MAT7 and IFS clusters (Supplementary Fig. 15). Some members of the families of three key genes of the formononetin biosynthesis pathway (IFS1, HIDH and HIOMT) (Supplementary Fig 11) were previously shown to be expressed at low to moderate levels in leaves of mature plants. Of those, the IFS1 gene (mRNA 15433) was expressed most highly (up to 362 RPKM under drought conditions), while HIDH (mRNAs 39329 and 5684) and HIOMT (mRNAs 15429 and 15438) had expression levels between 4 and 66 RPKM. The MAT7 and IF7GT gene families were expressed at lower levels (<15 RPKM).

Red clover has superior feeding value in terms of transfer of omega-3 fatty acids from ruminant feed to milk^25^, and reduced levels of proteolysis during wilting and ensiling of its biomass^26^. These properties have been linked to the prevalence of the enzyme polyphenol oxidase (PPO). This enzyme catalyses the conversion of endogenous di-phenols to quinones. The quinones can bind with proteins and reduce the speed of proteolysis and lipolysis in the rumen. In red clover PPO appears to form a cluster with three^26^ to seven^27^ members. We have identified five PPO genes in the red clover genome assembly (Supplementary Fig. 19). Four of them were highly similar to each other and different to PPO genes in related species. PPO1 and PPO2 genes were located 1 Mb apart on chromosome 6. A second copy of PPO1 was found on scaffold 8733, and PPO3 was located in scaffold 1247. The latter is also similar to mRNAs previously annotated as PPO4 and PPO5^27^. A further PPO gene, which is homologous to the three PPO genes present in *M. truncatula*, was found on chromosome 2 of red clover in a region conserved with chromosome 2 of *M. truncatula* (Supplementary Fig. 20). The existence of two single copy PPO genes in common bean, and six copies of each of these two genes in soybean is consistent with the hypothesis that the red clover genes in the chromosome 6 cluster are a result of duplication events, and that their homologues are missing in *M. truncatula*. Both the latter and *M. sativa*, in contrast to red clover, have little PPO activity^26^,^28^ implying that the PPO genes in these two species are inactive or lack a substrate.

### Linkage disequilibrium in a synthetic population of red clover

Linkage disequilibrium (LD) in a population determines the marker density required in genome wide association studies (GWAS) and for genomic selection (GS) in breeding programmes, as well as providing insight into population structure. The average LD at 100 Kb in the red clover variety Lea, a synthetic population with multiple parents, varied between 0.15 and 0.25 in the seven chromosomes (see Supplementary Table 5, and Supplementary Fig. 21 for graphs of LD decay, landscape and heatmaps). Given the marker density and genome size a QTL would be on average 76.5 Kb from the nearest marker. At this distance LD varied between 0.19 and 0.31 (Supplementary Table 5). Supplementary Fig. 5 shows that heterozygosity is close to equilibrium, which is consistent with the way in which synthetic populations are generated. The population was derived from three parental populations by polycrossing, but PCA analysis of the marker data was unable to separate the founder populations clearly, as the first two principal components accounted for only 4.3% of the variance (Supplementary Fig. 22).

## Discussion

This work provides a genome assembly on a pseudomolecule level of a highly heterozygous genome. The inbreeding depression and loss of viability associated with self-incompatibility of red clover^14^ has precluded the generation of inbred lines for sequencing purposes. The evidence from the comparison of several short-reads assemblers supported the use of Platanus as the best option to generate a high quality reference assembly in this heterozygous species (Supplementary Figs 1 and 2). We showed that there was no additional content in the other assemblies (Supplementary Table 2), and that our assembly is a significant step forward in comparison to the resources available to date.

The anchoring of a significant number of scaffolds, that contained at least half of the genes, allowed the spatial comparison of features between *M. truncatula* and red clover. We also estimated that the divergence of red clover and *M. truncatula* is comparable to the divergence of common bean and soybean. In general terms, the gene content, distribution, and length are conserved among legumes, which is relevant for translational agrigenomics. For example, the enzymes involved in the formononetin pathways are distributed in five clusters, the structure of which, are conserved between red clover and *M. truncatula*. Although the similar total content of repeats in *M. truncatula* and red clover is inconsistent with the hypothesis that outbreeding species have a higher potential for proliferation of transposable elements^21^, there are dramatic compositional differences between the two species (Fig. 1). The similar sized genomes, but contrasting breeding systems would appear to provide a good basis for comparison. It is possible that the compositional differences are associated with the different breeding systems, but perhaps other events such as chromosomal rearrangements are more closely associated with variation in repeat element composition.

The genotyping by sequencing analysis of the population based variety “Lea” has provided insight into the level of linkage disequilibrium in synthetic populations, which is one of the most common ways of generating new varieties in outbreeding forage crops. As expected there is no population structure, and low levels of linkage disequilibrium throughout the genome (Supplementary Fig 21). Nevertheless, the marker coverage would appear to be sufficient for meaningful studies of the genetics of complex traits, and genomics based breeding approaches, given that LD at the average marker distance was near 0.2 or above (Supplementary Table 5).

The unique feature of significant PPO activity in red clover in contrast to other forage legumes, notably *M. truncatula* and *M. sativa*^26^,^28^ would suggest that some of the genes in the cluster of PPO genes located on chromosome 6 and two unplaced scaffolds is responsible for the red clover PPO activity, rather than the PPO gene on chromosome 2, which is homologous to a PPO cluster on chromosome 2 in *M. truncatula* (Supplementary Fig. 19 and 20). However, previous RNASeq data show that PPO genes located on chromosome 2, 4, 6 and 7 were expressed at moderate levels in red clover leaves, and five of those PPO genes were upregulated after exposure to drought stress^10^. Other expression analyses in red clover suggest that the PPO4 gene is responsible for most of the activity in red clover mature leaf tissue^28^. This is most closely related to the PPO gene, described here as PPO3, which is located on scaffold 1247 (Supplementary Fig. 19). Two partial sequences from *T. repens*^28^ have the highest degree of similarity to the PPO gene on scaffold 1247 (PPO3), but have been described as PPO1 and PPO2^27^. Further experimental work is needed to establish the relative activity of the different PPO genes, and to what extent their activity is limited by substrate availability. The close relationship between red clover and *M. truncatula* will promote the translation of information from model species to forage crop, and this red clover assembly has facilitated analysis and mapping of pathways of particular importance for red clover nutritional quality.

## Methods

### Plant material and BAC libraries

The mapping population used in this work consisted of 188 genotypes of F_1_ progeny from a cross between a genotype of the variety Milvus and a genotype of the variety Britta. This population was generated initially to obtain material segregating for flowering time and a range of morphological characters. Three BAC libraries were created using high molecular weight DNA from a specific genotype of the Milvus variety (Milvus B). For one of the libraries the DNA was partially cut with the restriction endonuclease *Hin*dIII as described^27^. This library, named TP_MBa, consisted of more than 23000 clones of an average size of 125 Kb. Two other libraries were made, one cut with *Eco*RI (named TP_ABa) and one with *Bam*HI (TP_ABb) as described. They each consisted of 36864 clones, with similar average size inserts as the *Hin*dIII library. All three BAC libraries are available to the public from the Arizona Genomics Institute Resource Center (http://www.genome.arizona.edu/orders/).

### Physical map and BAC end sequencing

Using methods previously described, the three red clover BAC libraries were subjected to BAC clone end sequencing and BAC clone SNaPshot fingerprinting (FP)^29^. Specifically, we used 18432, 9216 and 9216 BAC clones from the libraries TP_MBa, TP_ABa, and TP_ABb, respectively, which together represented nearly 10x genome coverage. The output data provided the raw inputs of the genome frame to allow physical map construction, anchoring of genetic and physical maps to the *M. truncatula* reference sequence^3^, and for comparative analysis to other genome data sets. A *de novo* BAC clone physical map was assembled with the FP data using FPC software with the settings and parameters as previously described^29^.

### Genetic map construction

The F1 mapping population described above was used to generate the genetic map. A total of 153 markers, based on either single nucleotide polymorphisms (SNPs) or microsatellites (SSRs), were used. The SNPs were identified by amplicon sequencing of ESTs, either intron-spanning or within exons (as identified by BLAST hits to *M. truncatula*). Putative polymorphisms were first identified in the two parental genotypes, and then either sequenced in the whole population of 188 or genotyped by LGC using the KASPar methodology (http://www.lgcgroup.com). The SSR markers were either obtained from markers previously described^13^, or developed in this work from the BES sequences, by identification of repeats using the programme MISA (http://pgrc.ipk-gatersleben.de/misa/misa.html), followed by validation in a subset of the mapping population. Other markers were transferred from either *M. truncatula* or white clover from previous work^30^. The genetic map was constructed using JoinMap® 4^31^, and linkage groups were identified by the grouping module with a LOD score threshold of 4. The locus order was calculated with the regression mapping module with Kosambi’s mapping function for conversion of recombination frequency to cM, recombination frequency smaller than 0.4 and a LOD >1, goodness-of-fit Jump threshold for removal of loci = 5.0, number of added loci after which to perform a ripple = 1, and third round = Yes.

Amplification of genomic DNA was done in a 10 or 20 μl reaction volume depending upon whether amplification product was to be visualised by gel electrophoresis. Approximately 20 ng of genomic DNA was added to 1xAmpliTaq buffer, 0.2 mM dNTPs, 0.2μM forward and reverse primers and 1U of AmpliTaq DNA polymerase. The PCR amplifications were carried out in ABI 9700 (Applied Biosystems) with the following conditions: 10 min at 94 °C, then 35–40 cycles with 94 °C for 30 sec, Tm for 30 sec, and 72 °C for 1 min followed by a final extension at 72 °C for 7 min. The annealing temperature depended upon the individual primer pairs, but was typically 55–60 °C. The SSR amplifications involved fluorescent primers for subsequent analysis using an ABI 3730xl Genetic Analyzer (Applied Biosystems, Warrington, UK). Singleplex or multiplex reactions were run and analysed using GeneMapper v3.7 (Applied Biosystems, Warrington, UK). Amplicons for sequencing were cleaned to remove unincorporated primers and nucleotides with MicroCLEAN (Web Scientific, Crewe, UK) as described by the manufacturer, and prepared for sequencing according to ABI’s protocol for capillary sequencing.

### Library generation, sequencing and assembly

Eight different libraries (Supplementary Table 6, ENA accession PRJEB9186) were created from the same genotype of the Milvus variety (Milvus B) that was used for the BAC library construction, and were sequenced using Illumina HiSeq 2000 or MiSeq instruments at The Genome Analysis Center (TGAC, Norwich, UK). Four of the libraries encompassed a 150 bp single-end library, two paired-end libraries with insert sizes of 100 and 150 bp, plus one with 100 bp reads that overlapped in 25 bp. Additionally, four mate-pair libraries with insert lengths of 3, 5 and 7 Kb were also created to improve the scaffolding. Read quality was assessed, and contaminants and adaptors removed. Illumina Nextera MP reads were required to include a fragment of the adaptor to be used in the following steps^32^.

The two pair-end and two single-end shotgun libraries were assembled, and later scaffolded using mate-pairs libraries with four different insert lengths, using Platanus v1.2.1^17^, which is optimized for heterozygous genomes. Scaffolds shorter than 500 bp were filtered out. We used Kmer spectra analysis to compare the assemblies produced by different pipelines, as well as our final assembly with the previously published assembly. A K-mer spectrum is a representation of how many fixed-length words or K-mers (y-axis) appear a certain number of times or coverage (x-axis). We used 31mers in our plots. The K-mer counting was performed with Jellyfish^33^ and the comparison and plotting was performed with KAT, a tool developed at TGAC (https://github.com/TGAC/KAT). Further information can be found in the manual (https://documentation.tgac.ac.uk/display/KAT/KAT+Home). A new feature allows decomposing of the spectra into coloured components related to copy number, in order to represent the number of times that each K-mer appears in the final assembly.

For chromosome-scale pseudo-molecule construction, markers from the genetic maps were placed using BLAT^34^. Alignments that comprised >90% base-pair identity and >90% coverage were retained. We first placed the markers from the previously published genetic map^11^. Some markers aligned in more than one position, but were tagged and retained. Any single marker linking to a different linkage-group from that of the other markers placed in the same scaffold was removed. Secondly, we placed the markers from our genetic map with the same criteria. However, we discarded the new position information if it differed by more than 30 cM with the previous map. If not, we used the average value as the final position. Nine scaffolds were split into two because markers were anchored to different linkage-groups.

The BAC-end sequences were aligned to the hard-masked assembly using MegaBLAST^35^ with a requirement of >90% base-pair identity and >90% coverage. We filtered the physical sink contigs 0, 335 and 447, and quantified the number of alignments that supported each link between a scaffold and a physical contig. We accepted this if a) the scaffold always linked with the same physical contig (Unique links), b) more than 50% of the alignments were to the same contig, and c) there was a minimum number of total alignments (Dominant links), or the number of alignments to a physical contigs was significantly higher than to any other physical contig (Strong links). Most of the scaffolds with accepted links had been previously placed. In order to place a contig into a contiguous position we looked for any unplaced scaffold linking to the same contig as any previously placed scaffold. We used the EnsEMBL database and pipelines to construct the pseudo-molecules and reassign the coordinates of the features (genes, transcripts, exons, etc) to them. Each pseudo-molecule join was padded with a 10 Kbp gap.

### Genome annotation and comparative analysis

The annotation pipeline is represented in the Supplementary Fig. 23. Repetitive and low complexity regions of the scaffolds were masked using RepeatMasker^36^ based on self-alignments and homology with the RepBase public database and specific databases built with RepeatModeler^37^. LTR retrotransposons were detected by LTRharvest^38^. Repeat elements were classified with TEclass^39^ and RepeatClassifier^37^. The 5′ and 3′ ends of each LTR identified by LTRharvest were aligned with MUSCLE and used to calculate the nucleotide divergence rate with the Kimura-2 parameter using MEGA5^40^. The insertion time was estimated by assuming an average substitution rate of 1.3 × 10e-8, as in the common bean^5^ analysis.

*De novo* and genome guided *ab initio* transcripts were assembled from RNA-Seq reads^10^ using Trinity^41^ and Tophat/Cufflinks^42^, respectively. Additionally, exon-intron junctions were deduced from the mapping positions of the reads. Junctions supported by more than 3 reads were incorporated as evidence in Augustus. Assembled transcripts were aligned to the assembly and clustered in novel transcript models using PASA^43^. A high quality full-length non-redundant subset from the *ab initio* transcript models from PASA was used as a training set for Augustus. The proteins of the Fabaceae family in Uniprot and TrEMBL, and the transcripts annotated in the soybean, common bean, and *M. truncatula* genomes were aligned to the masked genome using Exonerate^44^.

Gene models were predicted by GeneID^45^ and SNAP^46^ in the masked version of the assembly, and by Augustus in the unmasked version of the assembly. Augustus builds the gene models to be compatible with the information from the alignments, the transcript models and junctions deduced from the RNA-Seq data, and annotated repeated regions and transposons. Additionally, alternative transcript models of a gene were reported for those incompatible with the provided alignments and transcript information.

Finally, RNA-Seq reads were mapped again using the guidance of the generated annotation by Tophat, a new set of transcripts assembled by Cufflinks and alternative splicing incorporated in the annotation by PASA. The annotated features were stored in an EnsEMBL database to allow visualisation and exportation.

The functional annotation of the proteome was done with an in-house pipeline (AnnotF) that compares the results of Blast2GO^47^ and InterProSCAN^48^. Clustering was based on eggNOG clusters. Genes within pathways were compared with RAxML 8.0.22^49^ (100 bootstrap replications). The proteomes of four Fabaceae species and *A. thaliana* were aligned, and single gene clusters filtered and concatenated after removing gaps using HAL^50^. A phylogenetic tree based on these data was built with MEGA6^51^ using Maximum-likelihood and 100 bootstrap replications. Divergence times in the phylogenetic tree were calculated with the RelTime method^52^ in MEGA6 using the divergence date between common bean and soybean as reference^6^. The gene density was calculated by dividing the total number of genes by the total length of each genome. Additionally, we calculated the same value for each interval of 10 Kb along the genome. The distribution of these values is reported as “mean number of genes in 10 Kb intervals”.

Syntenic blocks were identified with MUMMER^53^, analysed with SyMap^54^, using the default parameters, but with the requirement of 30 gene pairs to call a syntenic block, and plotted with Circos^55^. The syntenic gene pairs were aligned with MAFFT v 7^56^ and the alignments used to calculate the Kimura rates with MEGA6 in order to estimate the nucleotide divergence rates.

### Linkage disequilibrium

A population consisting of 86 genotypes from the red clover variety Lea (Graminor, Norway) was sown as part of a field experiment in Southern Norway. Genotyping by sequencing methodology^57^ was used to obtain SNP polymorphisms in the population. A minimum of 10 reads for each individual, and in the case of heterozygotes, a minimum of 2 reads of the minor allele, were required for SNP calling. After removing SNPs with missingness >0.20, or minor allele frequency <0.05, a total of 3942 SNPs were identified, of which 2161 were mapped onto the 7 pseudomolecules. LD heatmaps and associated plots were produced using R^58^ as described^59^.

## Additional Information

**Accession codes:** All shotgun read libraries (Supplementary Table 5) and the assembly are deposited in the European Nucleotide Archive (accession PRJEB9186).

**Data Availability**: The genome assembly and annotation can also be downloaded as individual files (http://dx.doi.org/10.5281/zenodo.17232) or browsed online (http://tgac-browser.tgac.ac.uk/trifolium_pratense). Accession numbers of the BAC end sequences are HR235466-298279.

**How to cite this article**: De Vega, J. J. *et al.* Red clover (*Trifolium pratense* L.) draft genome provides a platform for trait improvement. *Sci. Rep.* **5**, 17394; doi: 10.1038/srep17394 (2015).

## Supplementary Material

Supplementary Information

Supplementary Table 3

## References

[b1] Taylor N. L. & Quesenberry K. H. Red Clover Science. (Kluwer Academic Publishers, 1996).

[b2] Frame J., Charlton J. F. L. & Laidlaw A. S. Temperate Forage Legumes. (CAB International, 1998).

[b3] Young N. D. *et al.* The Medicago genome provides insight into the evolution of rhizobial symbioses. Nature 480, 520–524 (2011). 22089132 10.1038/nature10625 PMC3272368

[b4] Sato S. *et al.* Genome Structure of the Legume, Lotus japonicus. DNA Res. 15, 227–239 (2008). 18511435 10.1093/dnares/dsn008 PMC2575887

[b5] Schmutz J. *et al.* A reference genome for common bean and genome-wide analysis of dual domestications. Nat. Genet. 46, 707–713 (2014). 24908249 10.1038/ng.3008 PMC7048698

[b6] Schmutz J. *et al.* Genome sequence of the palaeopolyploid soybean. Nature 463, 178–183 (2010). 20075913 10.1038/nature08670

[b7] Varshney R. K. *et al.* Draft genome sequence of chickpea (Cicer arietinum) provides a resource for trait improvement. Nat. Biotech. 31, 240–246 (2013). 10.1038/nbt.2491 23354103

[b8] Varshney R. K. *et al.* Draft genome sequence of pigeonpea (Cajanus cajan), an orphan legume crop of resource-poor farmers. Nat. Biotech. 30, 83–89 (2012). 10.1038/nbt.2022 22057054

[b9] Yang S. S. *et al.* Using RNA-Seq for gene identification, polymorphism detection and transcript profiling in two alfalfa genotypes with divergent cell wall composition in stems. BMC Genomics 12, 199 (2011). 21504589 10.1186/1471-2164-12-199 PMC3112146

[b10] Yates S. *et al.* *De novo* assembly of red clover transcriptome based on RNA-Seq data provides insight into drought response, gene discovery and marker identification. BMC Genomics 15, 453 (2014). 24912738 10.1186/1471-2164-15-453 PMC4144119

[b11] Isobe S. *et al.* Construction of a consensus linkage map for red clover (Trifolium pratense L.). BMC Plant Biol. 9, 57 (2009). 19442273 10.1186/1471-2229-9-57 PMC2695442

[b12] Isobe S. N. *et al.* Comparative Genetic Mapping and Discovery of Linkage Disequilibrium Across Linkage Groups In White Clover (Trifolium repens L.). G3: Genes|Genomes|Genet. 2, 607–617 (2012). 10.1534/g3.112.002600 PMC3362943 22670230

[b13] Sato S. *et al.* Comprehensive Structural Analysis of the Genome of Red Clover (Trifolium pratense L.). DNA Res. 12, 301–364 (2005). 16769692 10.1093/dnares/dsi018

[b14] Taylor N. L. Stability of S Alleles in a Doublecross Hybrid of Red Clover1. Crop Sci. 22, 1222–1225 (1982).

[b15] Ištvánek J., Jaroš M., Křenek A. & Řepková J. Genome assembly and annotation for red clover (Trifolium pratense; Fabaceae). Am. J. Bot. 101, 327–337 (2014). 24500806 10.3732/ajb.1300340

[b16] Ellison N. W., Liston A., Steiner J. J., Williams W. M. & Taylor N. L. Molecular phylogenetics of the clover genus (Trifolium--Leguminosae). Mol. Phylogenet. Evol. 39, 688–705 (2006). 16483799 10.1016/j.ympev.2006.01.004

[b17] Kajitani R. *et al.* Efficient *de novo* assembly of highly heterozygous genomes from whole-genome shotgun short reads. Genome Res. 24, 1384–1395 (2014). 24755901 10.1101/gr.170720.113 PMC4120091

[b18] Simpson J. T. *et al.* ABySS: A parallel assembler for short read sequence data. Genome Res. 19, 1117–1123 (2009). 19251739 10.1101/gr.089532.108 PMC2694472

[b19] Li R. *et al.* SOAP2: an improved ultrafast tool for short read alignment. Bioinformatics 25, 1966–1967, (2009). 19497933 10.1093/bioinformatics/btp336

[b20] Parra G., Bradnam K., Ning Z., Keane T. & Korf I. Assessing the gene space in draft genomes. Nucleic Acids Res. 37, 289–297 (2009). 19042974 10.1093/nar/gkn916 PMC2615622

[b21] Lavin M., Herendeen P. S. & Wojciechowski M. F. Evolutionary Rates Analysis of Leguminosae Implicates a Rapid Diversification of Lineages during the Tertiary. Syst. Biol. 54, 575–594 (2005). 16085576 10.1080/10635150590947131

[b22] Adams N. R. Detection of the effects of phytoestrogens on sheep and cattle. J. Anim. Sci. 73, 1509–1515 (1995). 7665383 10.2527/1995.7351509x

[b23] Moorby J. M., Fraser M. D., Theobald V. J., Wood J. D. & Haresign W. The effect of red clover formononetin content on live-weight gain, carcass characteristics and muscle equol content of finishing lambs. Anim. Sci. 79, 303–313 (2004).

[b24] Tsao R., Papadopoulos Y., Yang R., Young J. C. & McRae K. Isoflavone Profiles of Red Clovers and Their Distribution in Different Parts Harvested at Different Growing Stages. J. Agric. Food Chem. 54, 5797–5805 (2006). 16881680 10.1021/jf0614589

[b25] Dewhurst R. J., Fisher W. J., Tweed J. K. S. & Wilkins R. J. Comparison of Grass and Legume Silages for Milk Production. 1. Production Responses with Different Levels of Concentrate. J. Dairy Sci. 86, 2598–2611 (2003). 12939084 10.3168/jds.S0022-0302(03)73855-7

[b26] Sullivan M. L., Hatfield R. D., Thoma S. L. & Samac D. A. Cloning and Characterization of Red Clover Polyphenol Oxidase cDNAs and Expression of Active Protein in Escherichia coli and Transgenic Alfalfa. Plant Physiol. 136, 3234–3244 (2004). 15466227 10.1104/pp.104.047449 PMC523382

[b27] Winters A. *et al.* Identification of an extensive gene cluster among a family of PPOs in Trifolium pratense L. (red clover) using a large insert BAC library. BMC Plant Biol. 9, 94 (2009). 19619287 10.1186/1471-2229-9-94 PMC3224681

[b28] Webb K. J., Cookson A., Allison G., Sullivan M. L. & Winters A. L. Gene Expression Patterns, Localization, and Substrates of Polyphenol Oxidase in Red Clover (Trifolium pratense L.). J. Agric. Food Chem. 61, 7421–7430 (2013). 23790148 10.1021/jf401122d

[b29] Soderlund C., Humphray S., Dunham A. & French L. Contigs built with fingerprints, markers and FPCV4.7. Genome Res. 10, 1772–1787 (2000). 11076862 10.1101/gr.gr-1375r PMC310962

[b30] Zainol R. Molecular Genetic Analysis of Key Traits in Red Clover (Trifolium pratense L.). PhD thesis, Aberystwyth University, (2008).

[b31] JoinMap® 4, Software for the calculation of genetic linkage maps in experimental populations (Wageningen, Netherlands, 2006).

[b32] Leggett R. M., Clavijo B. J., Clissold L., Clark M. D. & Caccamo M. NextClip: an analysis and read preparation tool for Nextera Long Mate Pair libraries. Bioinformatics 30, 566–568 (2014). 24297520 10.1093/bioinformatics/btt702 PMC3928519

[b33] Marçais G. & Kingsford C. A fast, lock-free approach for efficient parallel counting of occurrences of k-mers. Bioinformatics 27, 764–770 (2011). 21217122 10.1093/bioinformatics/btr011 PMC3051319

[b34] Kent W. J. BLAT—The BLAST-Like Alignment Tool. Genome Res. 12, 656–664 (2002). 11932250 10.1101/gr.229202 PMC187518

[b35] Camacho C. *et al.* BLAST+: architecture and applications. BMC Bioinformatics 10, 421 (2009). 20003500 10.1186/1471-2105-10-421 PMC2803857

[b36] RepeatMasker Open-3.0 v.http://www.repeatmasker.org (1996–2010).

[b37] RepeatModeler Open-1.0 v.http://www.repeatmasker.org (2008–2010).

[b38] Ellinghaus D., Kurtz S. & Willhoeft U. LTRharvest, an efficient and flexible software for *de novo* detection of LTR retrotransposons. BMC Bioinformatics 9, 18 (2008). 18194517 10.1186/1471-2105-9-18 PMC2253517

[b39] Abrusán G., Grundmann N., DeMester L. & Makalowski W. TEclass—a tool for automated classification of unknown eukaryotic transposable elements. Bioinformatics 25, 1329–1330 (2009). 19349283 10.1093/bioinformatics/btp084

[b40] Tamura K. *et al.* MEGA5: Molecular Evolutionary Genetics Analysis Using Maximum Likelihood, Evolutionary Distance, and Maximum Parsimony Methods. Mol. Biol. Evol. 28, 2731–2739 (2011). 21546353 10.1093/molbev/msr121 PMC3203626

[b41] Grabherr M. G. *et al.* Full-length transcriptome assembly from RNA-Seq data without a reference genome. Nat. Biotech. 29, 644–652 (2011). 10.1038/nbt.1883 PMC3571712 21572440

[b42] Trapnell C. *et al.* Differential gene and transcript expression analysis of RNA-seq experiments with TopHat and Cufflinks. Nat. Protocols 7, 562–578 (2012). 22383036 10.1038/nprot.2012.016 PMC3334321

[b43] Haas B. J. *et al.* Improving the Arabidopsis genome annotation using maximal transcript alignment assemblies. Nucleic Acids Res. 31, 5654–5666 (2003). 14500829 10.1093/nar/gkg770 PMC206470

[b44] Slater G. & Birney E. Automated generation of heuristics for biological sequence comparison. BMC Bioinformatics 6, 31 (2005). 15713233 10.1186/1471-2105-6-31 PMC553969

[b45] Guigo R., Knudsen S., Drake N. & Smith T. F. Prediction of gene structure. J. Mol. Biol. 226, 141–157 (1992). 1619647 10.1016/0022-2836(92)90130-c

[b46] Korf I. Gene finding in novel genomes. BMC Bioinformatics 5, 59 (2004). 15144565 10.1186/1471-2105-5-59 PMC421630

[b47] Conesa A. *et al.* Blast2GO: a universal tool for annotation, visualization and analysis in functional genomics research. Bioinformatics 21, 3674–3676 (2005). 16081474 10.1093/bioinformatics/bti610

[b48] Jones P. *et al.* InterProScan 5: genome-scale protein function classification. Bioinformatics 30, 1236–1240 (2014). 24451626 10.1093/bioinformatics/btu031 PMC3998142

[b49] Stamatakis A. RAxML version 8: a tool for phylogenetic analysis and post-analysis of large phylogenies. Bioinformatics 30, 1312–1313, (2014). 24451623 10.1093/bioinformatics/btu033 PMC3998144

[b50] Robbertse B., Yoder R. J., Boyd A., Reeves J. & Spatafora J. W. Hal: an Automated Pipeline for Phylogenetic Analyses of Genomic Data. PLoS Currents 3, RRN1213 (2011). 21327165 10.1371/currents.RRN1213 PMC3038436

[b51] Tamura K., Stecher G., Peterson D., Filipski A. & Kumar S. MEGA6: Molecular Evolutionary Genetics Analysis Version 6.0. Mol. Biol. Evol. 30, 2725–2729 (2013). 24132122 10.1093/molbev/mst197 PMC3840312

[b52] Tamura K. *et al.* Estimating divergence times in large molecular phylogenies. Proc. Natl. Acad. Sci. USA 109, 19333–19338 (2012). 23129628 10.1073/pnas.1213199109 PMC3511068

[b53] Kurtz S. *et al.* Versatile and open software for comparing large genomes. Genome Biol. 5, R12 (2004). 14759262 10.1186/gb-2004-5-2-r12 PMC395750

[b54] Soderlund C., Nelson W., Shoemaker A. & Paterson A. SyMAP: A system for discovering and viewing syntenic regions of FPC maps. Genome Res. 16, 1159–1168 (2006). 16951135 10.1101/gr.5396706 PMC1557773

[b55] Krzywinski M. *et al.* Circos: An information aesthetic for comparative genomics. Genome Res. 19, 1639–1645 (2009). 19541911 10.1101/gr.092759.109 PMC2752132

[b56] Katoh K. & Standley D. M. MAFFT Multiple Sequence Alignment Software Version 7: Improvements in Performance and Usability. Mol. Biol. Evol. 30, 772–780 (2013). 23329690 10.1093/molbev/mst010 PMC3603318

[b57] Elshire R. J. *et al.* A Robust, Simple Genotyping-by-Sequencing (GBS) Approach for High Diversity Species. PLoS ONE 6, e19379 (2011). 21573248 10.1371/journal.pone.0019379 PMC3087801

[b58] Core Team R. (2013). R: A language and environment for statistical computing. R Foundation for Statistical Computing, Vienna, Austria. URL http://www.R-project.org/.

[b59] Wang L., Sorensen P., Janss L., Ostersen T. & Edwards D. Genome-wide and local pattern of linkage disequilibrium and persistence of phase for 3 Danish pig breeds. BMC Genet. 14, 115 (2013). 24304600 10.1186/1471-2156-14-115 PMC4235030

