# Supplementary material for: Red clover (Trifolium pratense L.) draft genome provides a platform for trait improvement
Source: Sci Rep. 2015 Nov 30;5:17394. doi: 10.1038/srep17394 (PMC4663792; doi:10.1038/srep17394)
Supplement: Supplementary Information [file srep17394-s1.pdf]

Red clover (*Trifolium pratense* L.) draft genome provides a platform for trait improvement

Jose J. De Vega[1,2*], Sarah Ayling[1], Matthew Hegarty[2], Dave Kudrna[3], Jose L. Goicoechea[3], Åshild Ergon[4], Odd A. Rognli[4], Charlotte Jones[2], Martin Swain[2], Rene Geurts[5], Chunting Lang[5], Klaus F. X. Mayer[6], Stephan Rössner[6], Steven Yates[2,7], Kathleen J. Webb[2], Iain S. Donnison[2], Giles E. D. Oldroyd[8], Rod A. Wing[3], Mario Caccamo[1], Wayne Powell[2,9], Michael T. Abberton[2,10], Leif Skøt[2*]

[1]The Genome Analysis Centre, Norwich Research Park, Norwich, NR4 7UH, UK

[2]Institute of Biological, Environmental and Rural Sciences, Aberystwyth University, Gogerddan, Aberystwyth, Ceredigion SY23 3EB, UK

[3]Arizona Genomics Institute, School of Plant Sciences, University of Arizona, Tucson AZ 85721, USA

[4]Norwegian University of Life Sciences, Department of Plant Sciences, N-1432, Ås, Norway

[5]Laboratory of Molecular Biology, Department of Plant Science, Wageningen University, Droevendaalsesteeg 1, 6708PB Wageningen, The Netherlands

[6]MIPS/Institute for Bioinformatics and Systems Biology, Helmholtz Center Munich, Ingolstädter Landstrasse 1, Neuherberg, Germany

[7]Forage Crop Genetics, Institute of Agricultural Sciences, ETH Zurich, CH-8092, Zurich, Switzerland

[8]Department of Disease and Stress Biology, John Innes Centre, Norwich NR4 7UH, UK

[9]CGIAR Consortium Office 1000, Avenue Agropolis, F-34394, Montpellier, Cedex 5, France

[10]International Institute of Tropical Agriculture (IITA), PMB 5320, Oyo Road, Ibadan, Nigeria

*e-mail: Jose.DeVega@tgac.ac.uk; lfs@aber.ac.uk

Supplementary Material

- Figures 1-23
- Tables 1-6
- Supplementary References

Supplementary Figures

Figure 1. Cumulative length (y-axis) plot of the red clover scaffolds sorted by length (x-axis) in the final assembly produced with Platanus 1.2.1 (Black line) and an alternative assembly assembled with ABySS and scaffolds with SOAP2 (Red line). (Top) All the scaffolds, and (Bottom) the 20000 longest scaffolds.

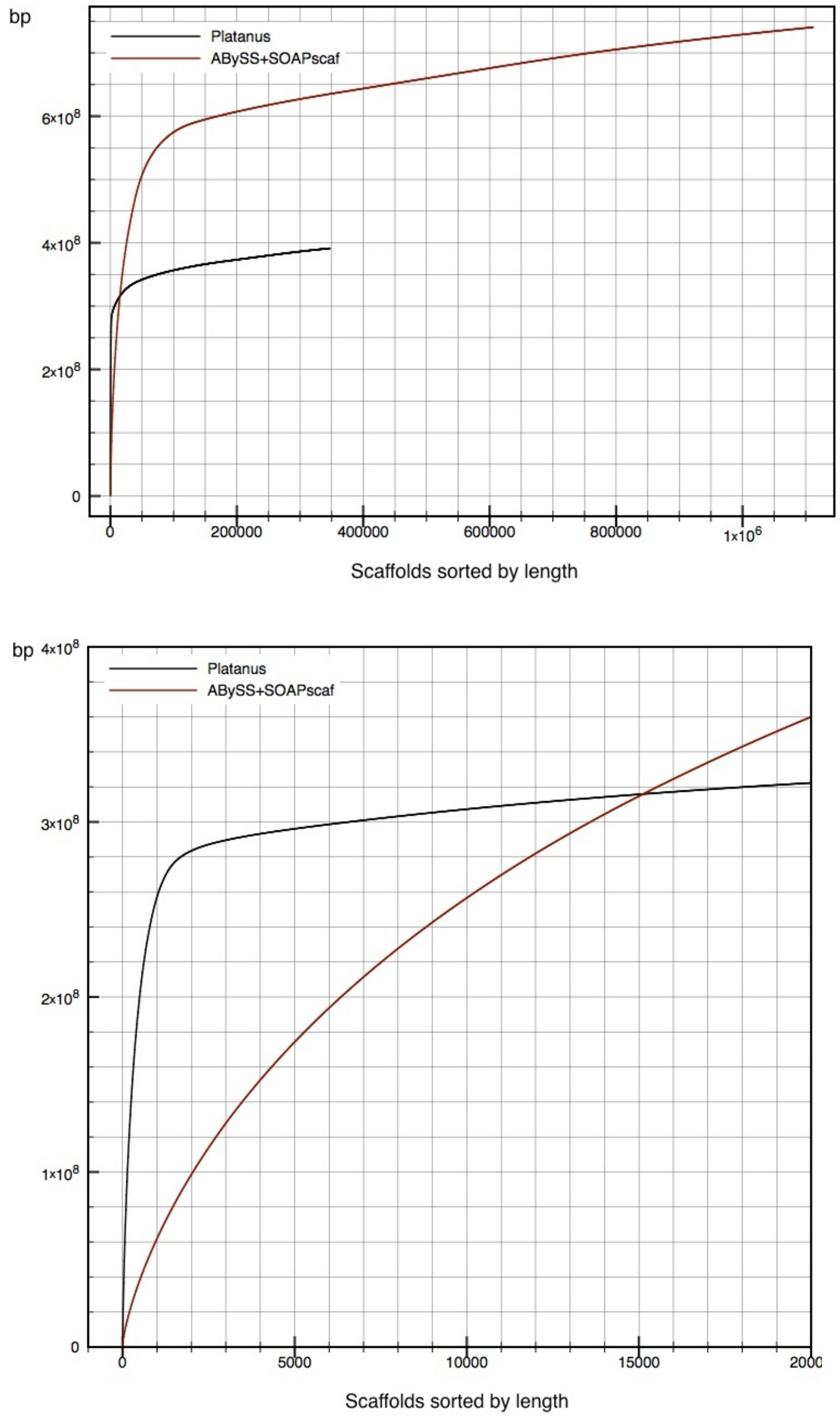

Figure 2. **Comparison of the Kmer spectra of four assembly strategies.** The K-mer spectra of the paired-end and single-end reads versus the different output of the assemblers. The area under the curve of the Kmer spectra has been coloured according to the number of times that such K-mers appear in the assembly: none in back, once in red, twice in orange, etc.

Platanus (Final reference)

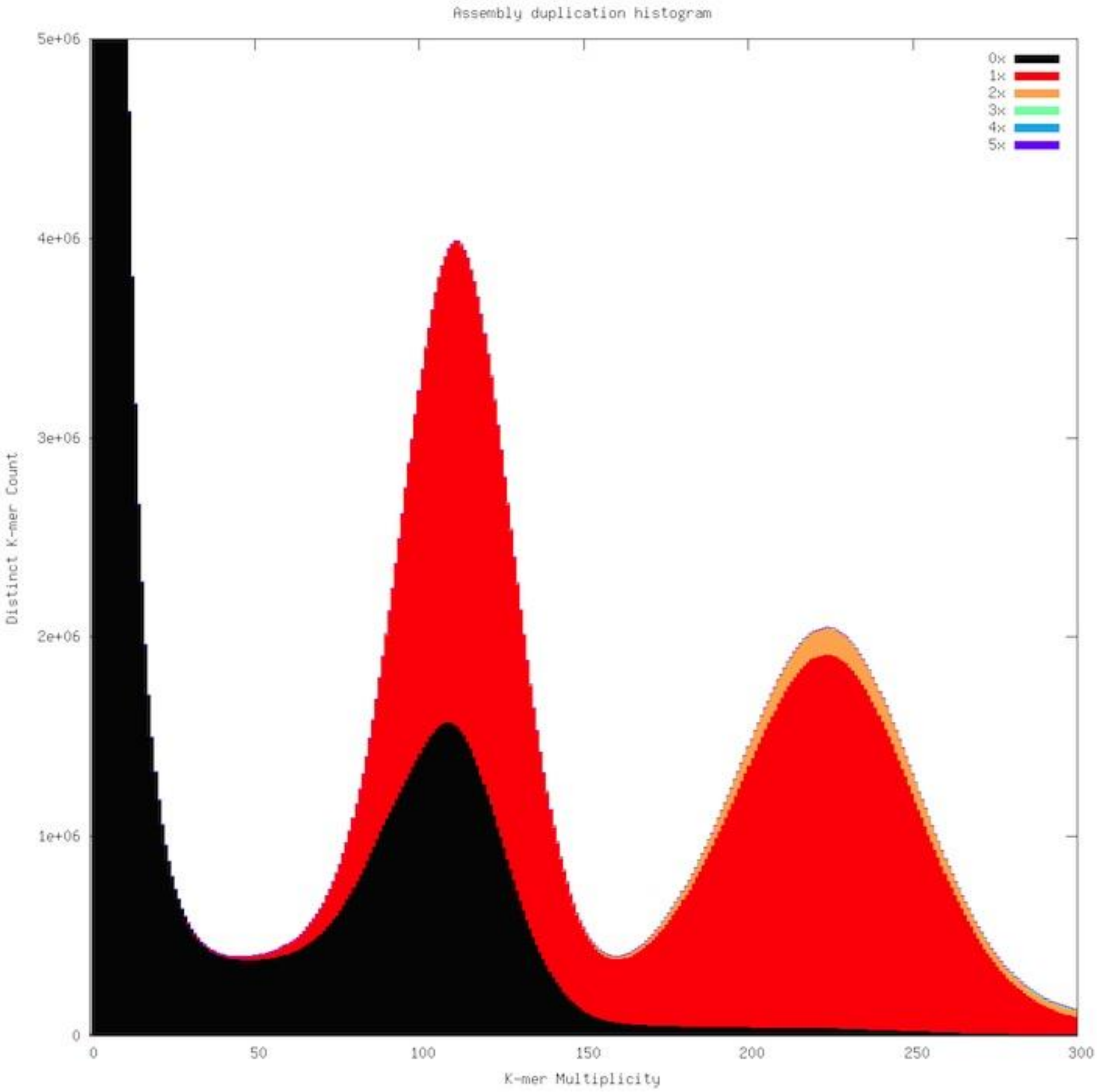

ABySS

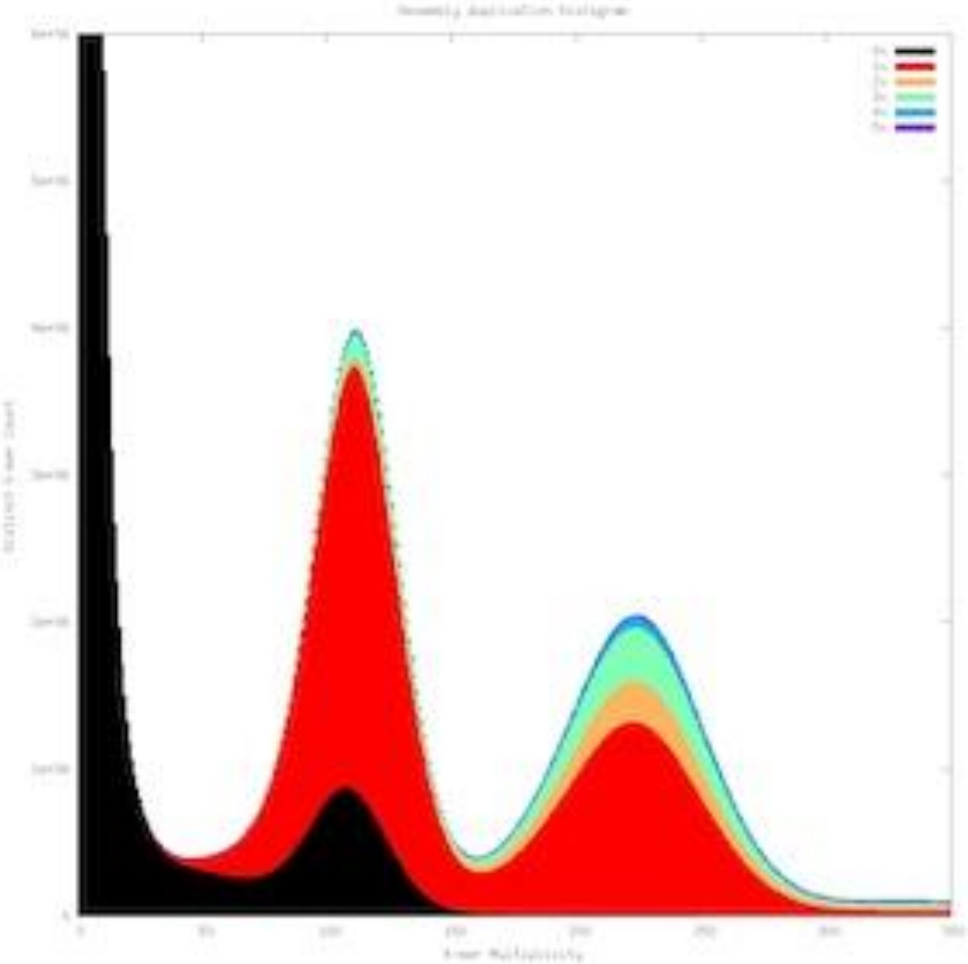

SOAP2

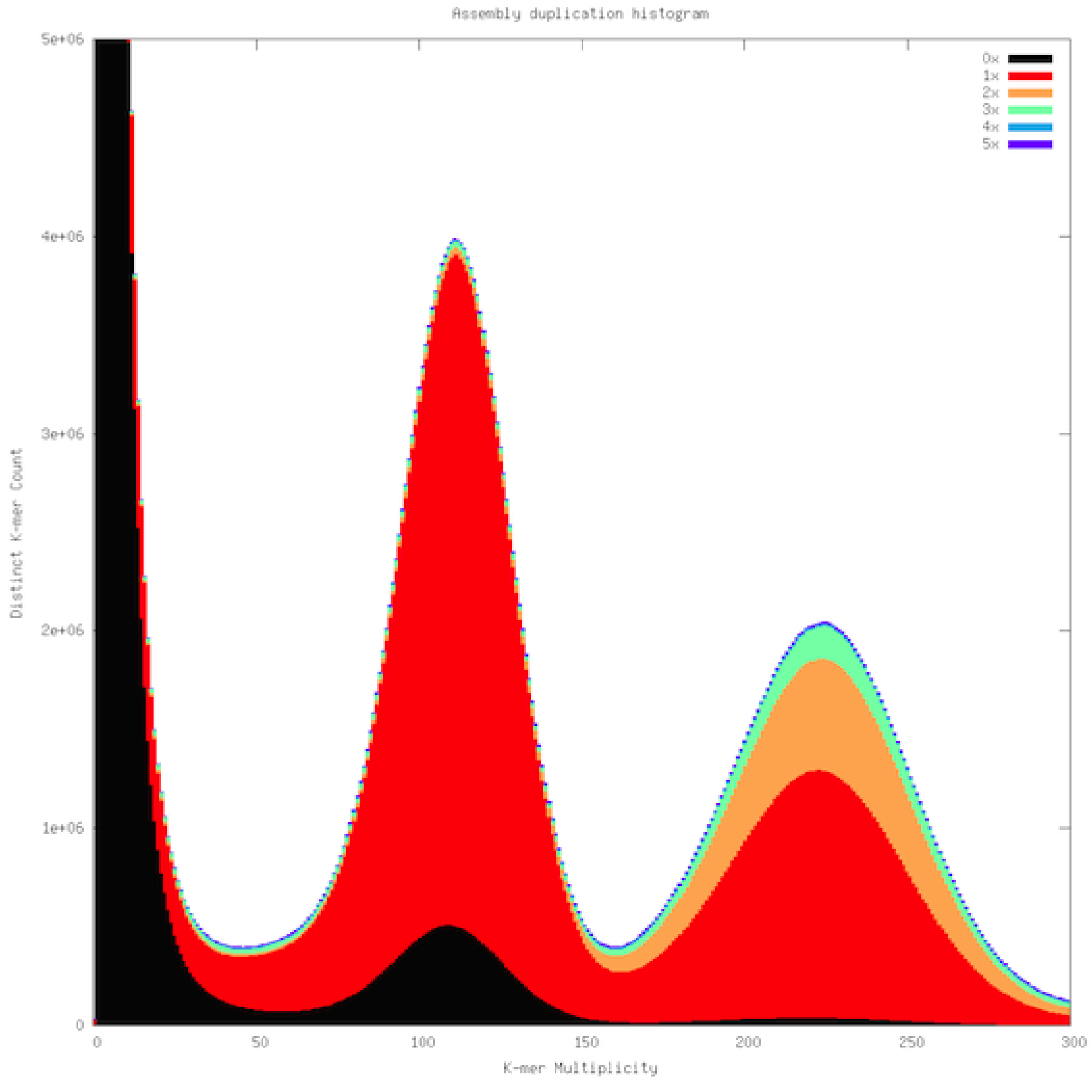

Assembly duplication histogram
Distinct K-mer Count
K-mer Multiplicity
0x
1x
2x
3x
4x
5x
5e+06
4e+06
3e+06
2e+06
1e+06
0
0
50
100
150
200
250
300

SOAP2 + GAPCLOSER

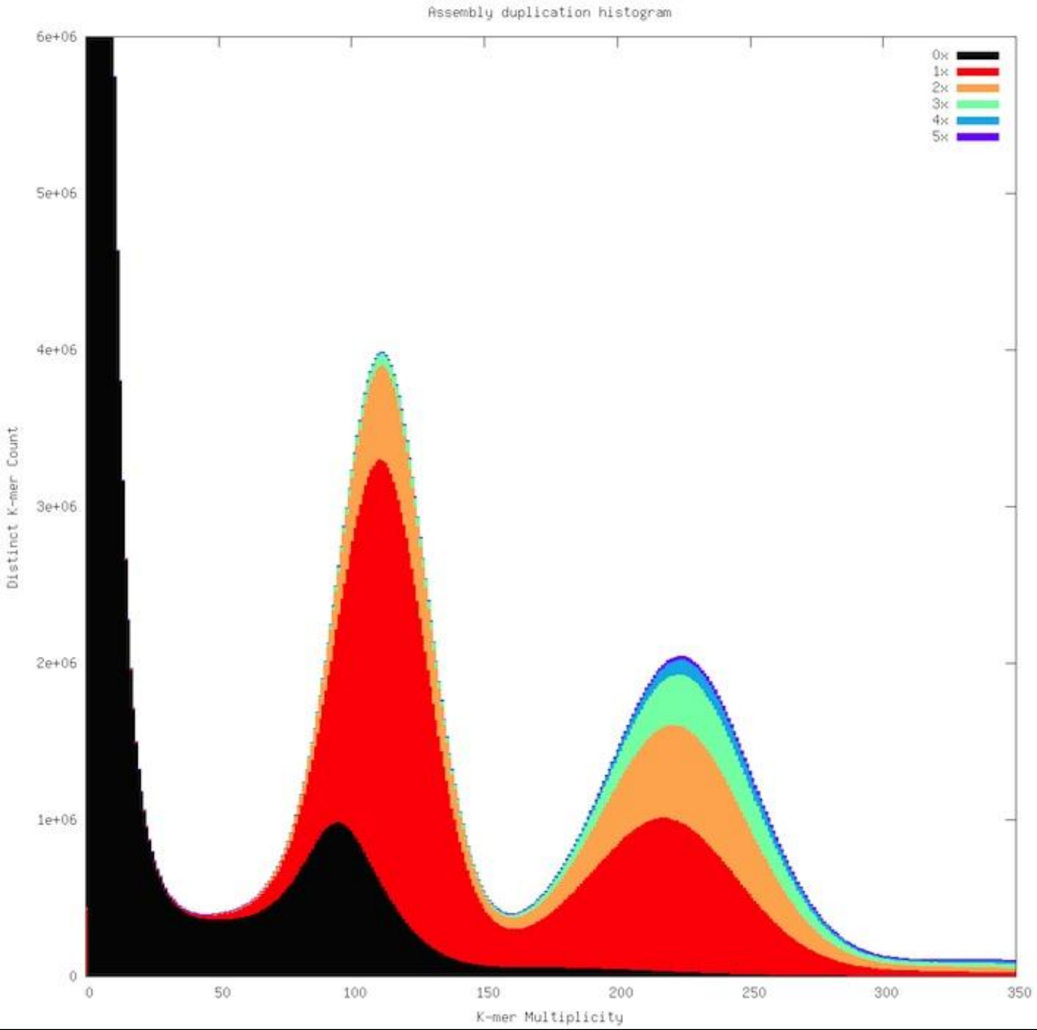

Figure 3. K-mer spectra of the red clover assembly described previously[1] (see also Results section).

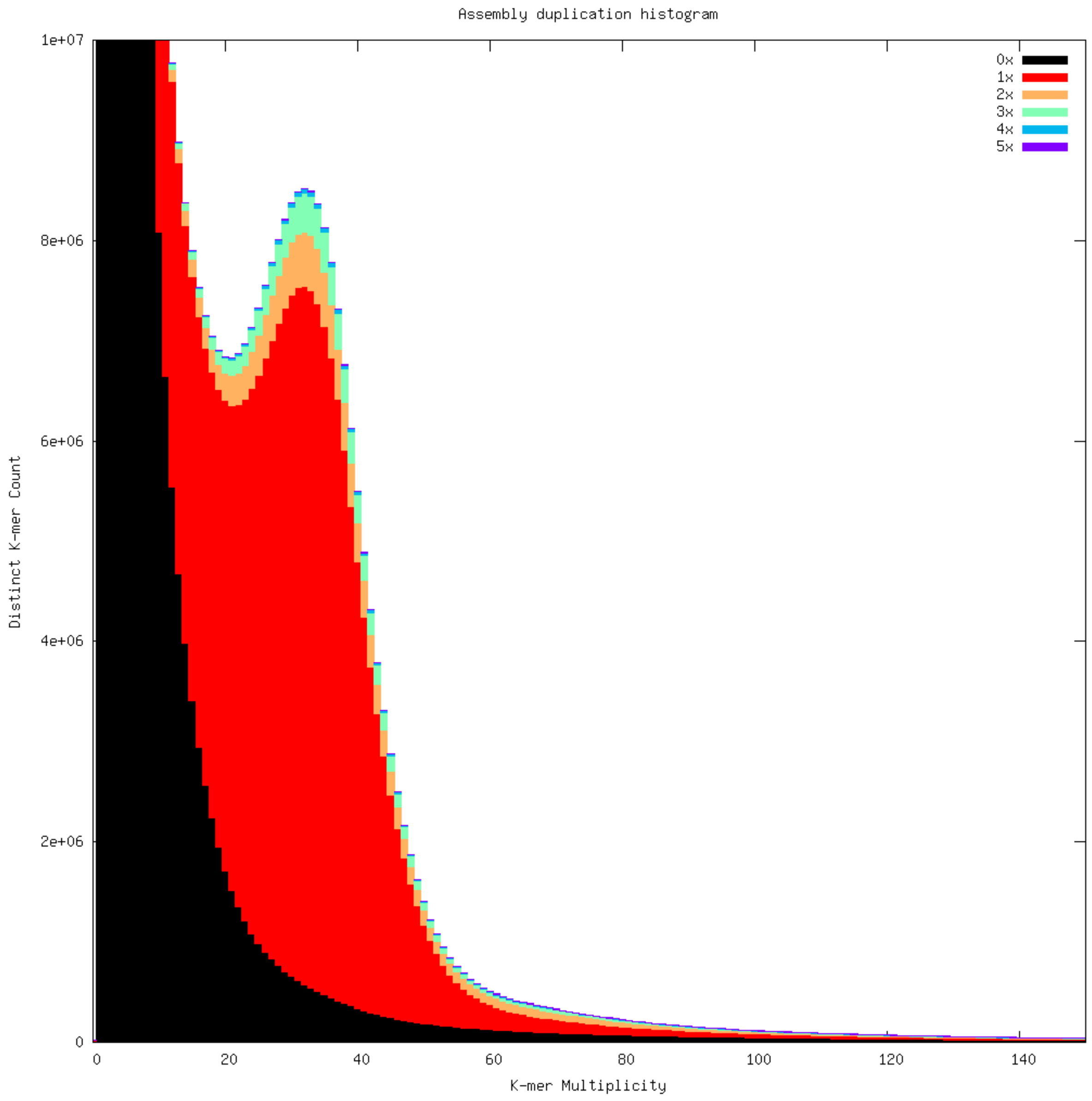

Figure 4. Genetic map of red clover Milvus x Britta F1 population. Genetic distances on the left of each linkage group (LG) are in cM.

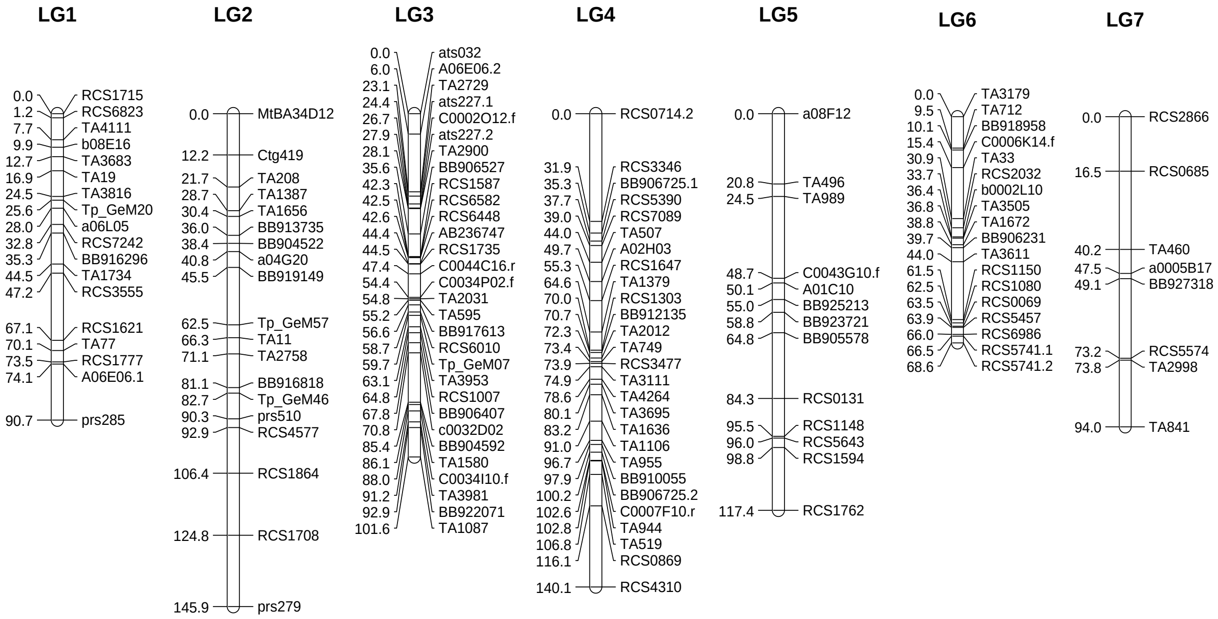

Figure 5. Landscapes of the red clover chromosomes. The landscapes represent the proportion (0-1) of content along the chromosome in 10Kb intervals of each of the elements in different categories. Top panel: Density of various types of repetitive elements. Circular symbols represent genetic markers. Green line is gene density per Mb. So, a density of 0.5 means that the gene content (exons and introns) occupied 500 Kb of each 1 Mb in each window. Bottom panel: Expected minus Observed Heterozygosity.

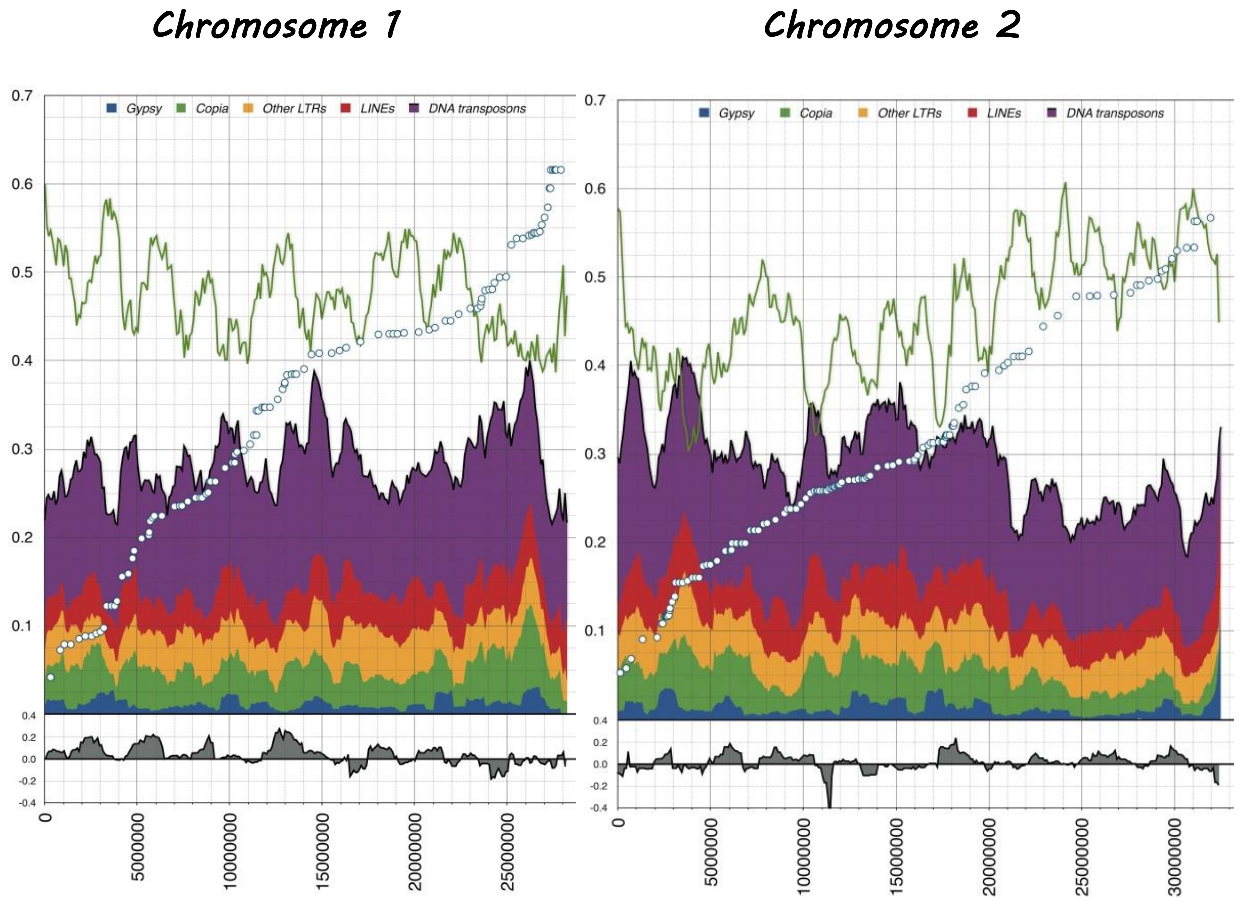

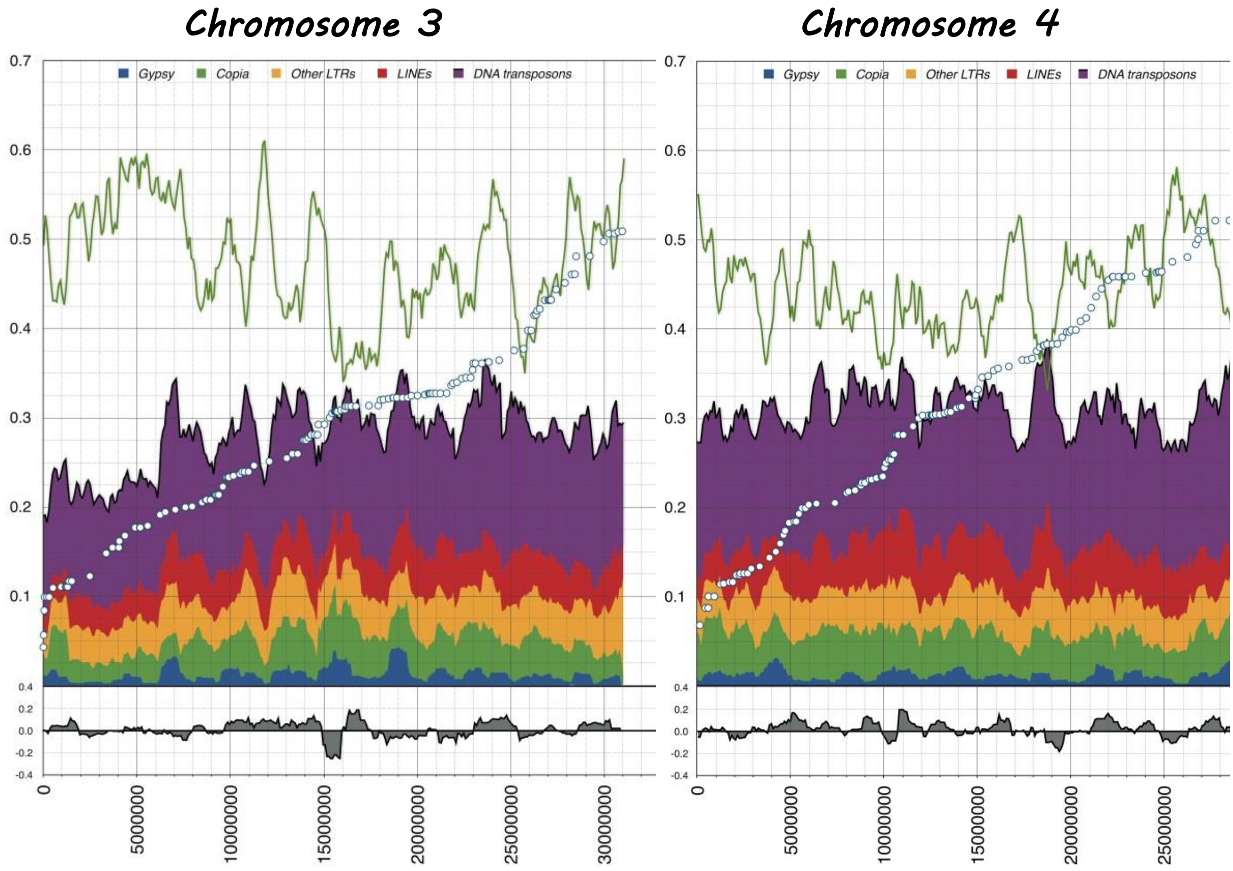
Chromosome 3
Chromosome 4
Gypsy
Copia
Other LTRs
LINEs
DNA transposons
0.7
0.6
0.5
0.4
0.3
0.2
0.1
0.4
0.2
0.0
-0.2
-0.4
0
5000000
10000000
15000000
20000000
25000000
30000000

De Vega *et al.* (2015). Red clover (*Trifolium pratense* L.) draft genome provides a platform for trait improvement.

Chromosome 5

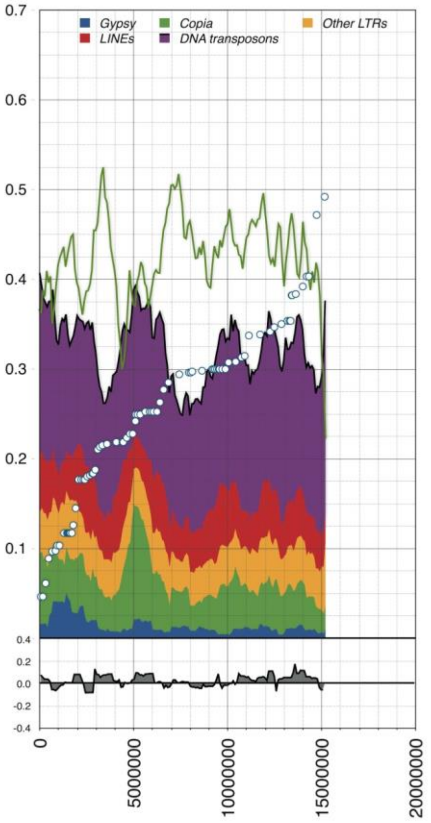

Chromosome 6

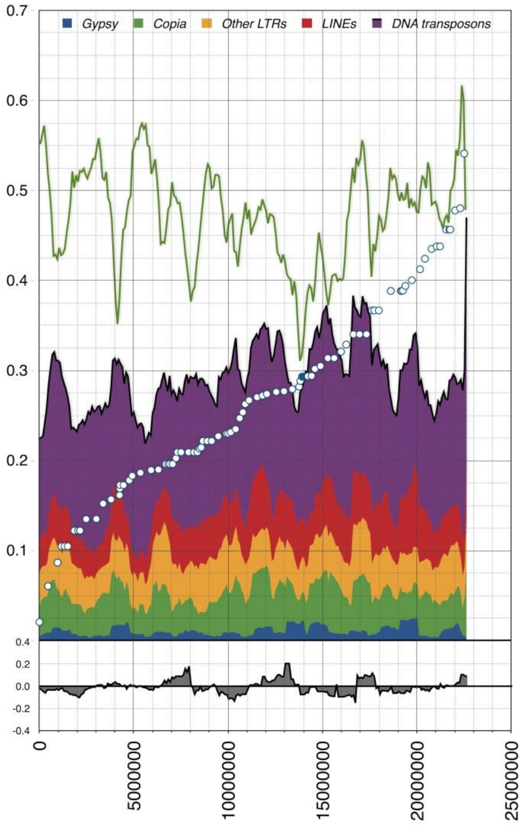

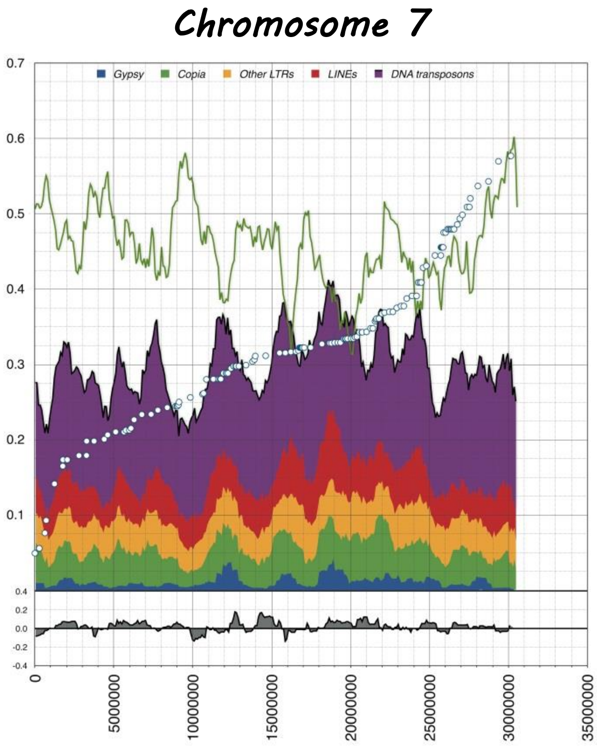

Chromosome 7
Gypsy
Copia
Other LTRs
LINEs
DNA transposons
0.7
0.6
0.5
0.4
0.3
0.2
0.1
0.4
0.2
0.0
-0.2
-0.4
0
5000000
10000000
15000000
20000000
25000000
30000000
35000000

Figure 6. Comparison of gene features. Length and frequency of the features (genes, transcripts, CDS and exons/introns) in the red clover (Tp), common bean (Pv), *M. truncatula* (Mt), *L. japonicus* (Lj), soybean (Gm) and *A. thaliana* (At) genomes.

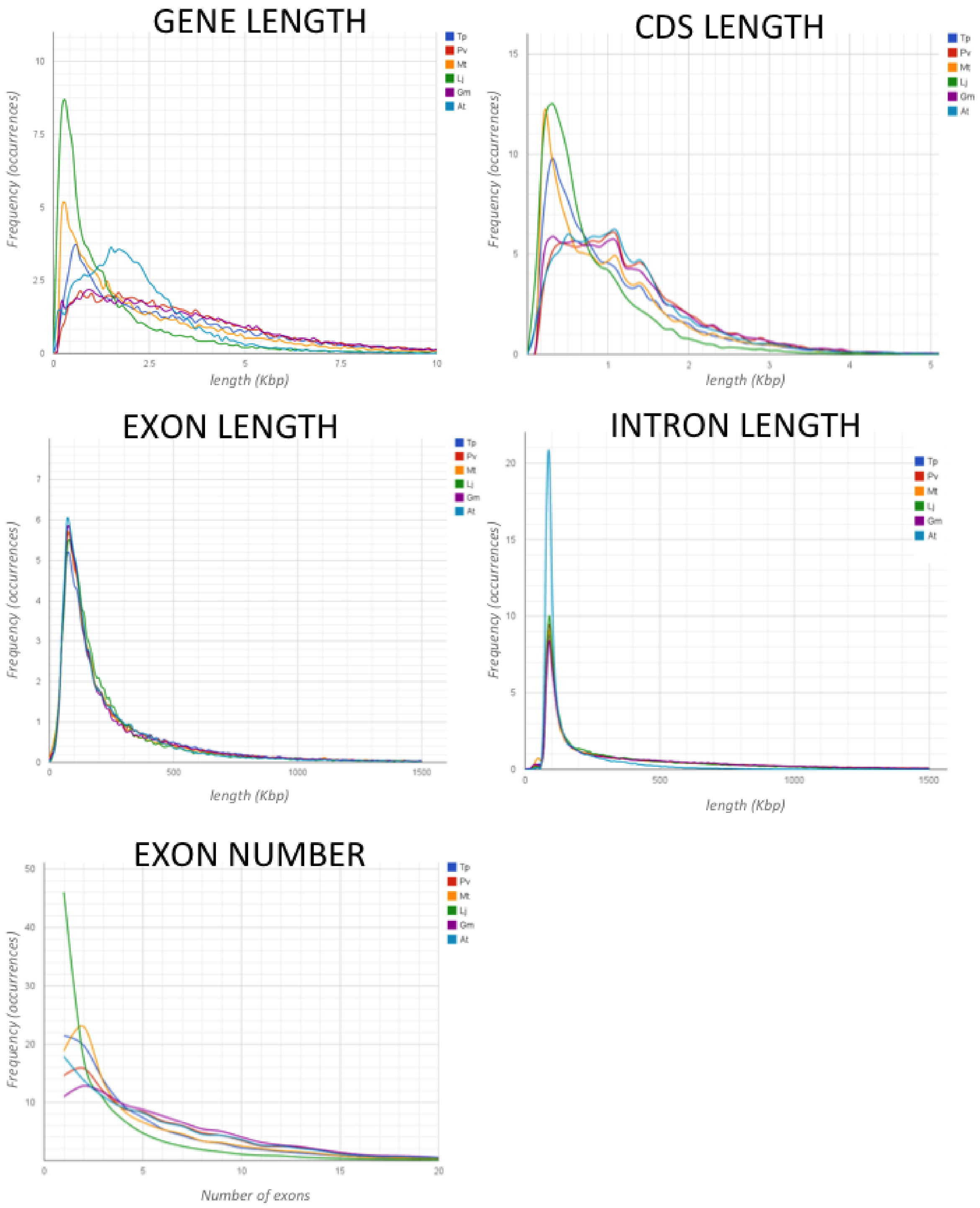

Figure 7. GO terms over-represented in red clover gene clusters that are expanded in comparison with *M. truncatula*. (A) Scatter-plot that represents the number of genes in each cluster in *M. truncatula* (X-axis) and red clover (Y-axis). A cluster is expanded when the number of members in red clover is at least twice the number of members in *M. truncatula*. (B) GO-terms overrepresented and proportion of sequences observed in the expanded clusters (blue columns) versus expected/total (red columns). (C&D) Treemaps with the biological process GO terms (C) and Molecular activity GO terms (D). The area is proportional to the enrichment, so bigger areas represent highly overrepresented functions in red clover.

7A

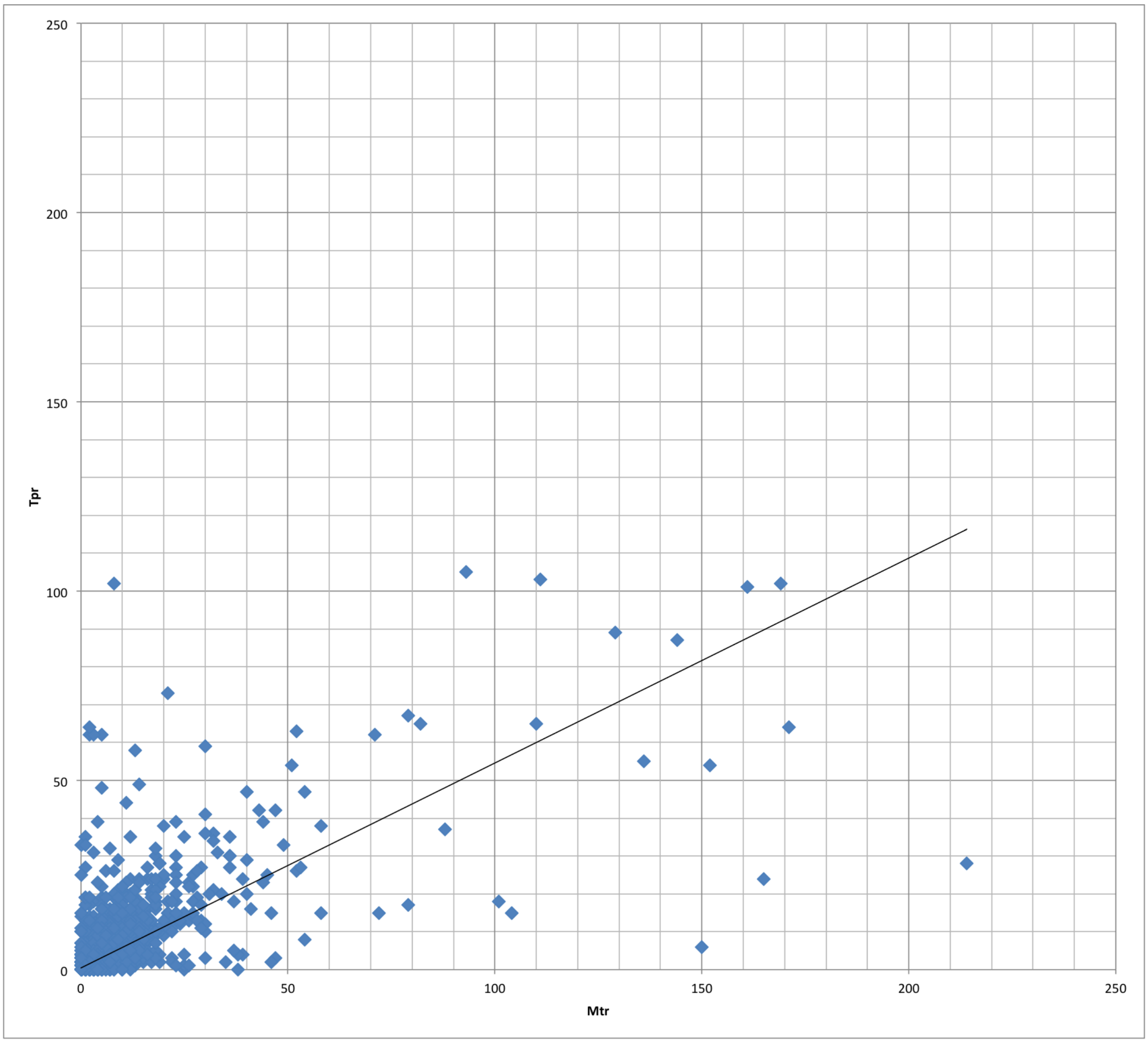

7B

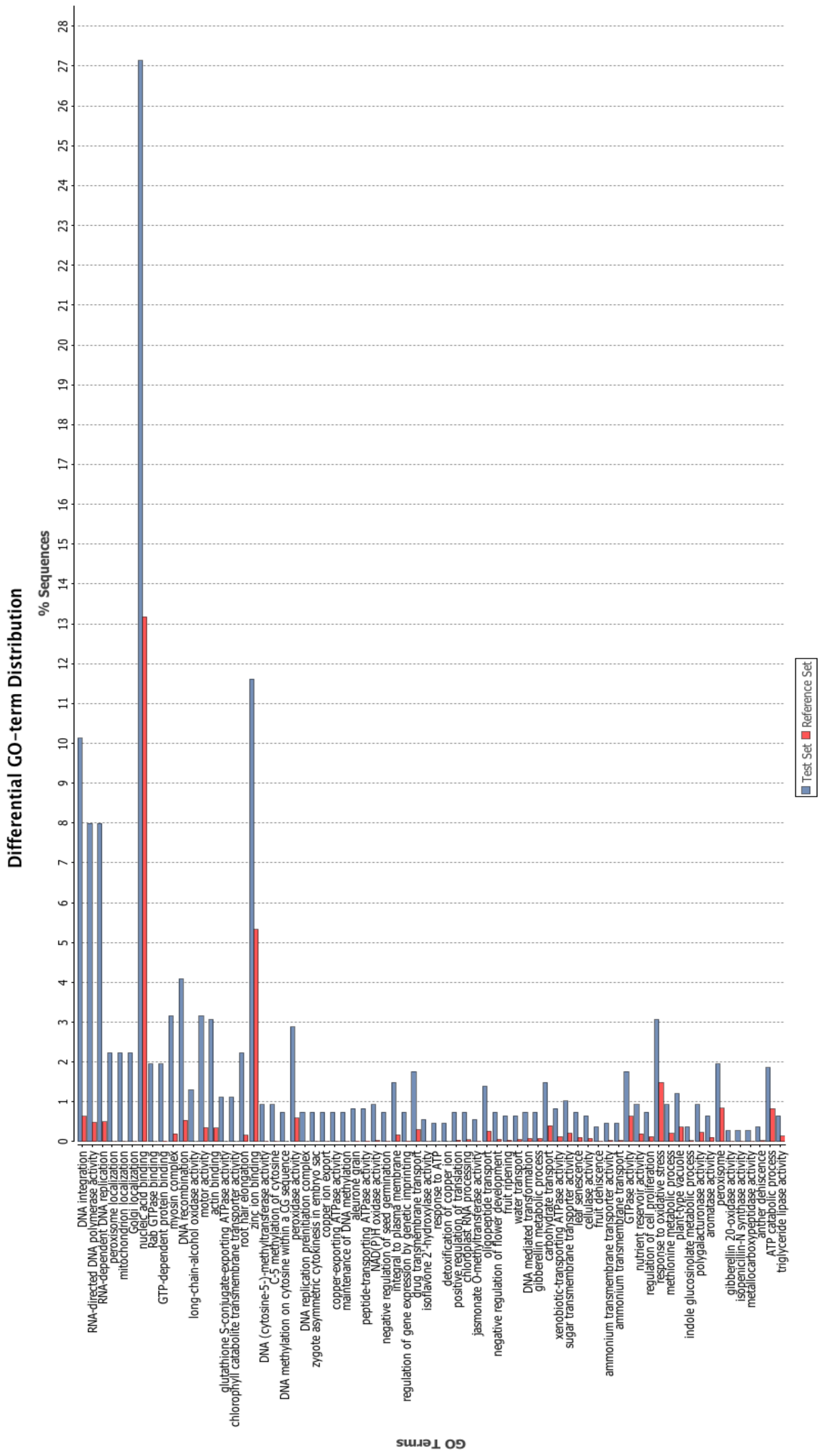
Differential GO-term Distribution
% Sequences
0
1
2
3
4
5
6
7
8
9
10
11
12
13
14
15
16
17
18
19
20
21
22
23
24
25
26
27
28
Test Set
Reference Set
GO Terms
DNA integration
RNA-directed DNA polymerase activity
RNA-dependent DNA replication
peroxisome localization
mitochondrion localization
Golgi localization
nucleic acid binding
Rab GTPase binding
GTP-dependent protein binding
myosin complex
DNA recombination
long-chain-alcohol oxidase activity
motor activity
actin binding
glutathione S-conjugate-exporting ATPase activity
chlorophyll catabolite transmembrane transporter activity
root hair elongation
zinc ion binding
DNA (cytosine-5-)-methyltransferase activity
C-5 methylation of cytosine
DNA methylation on cytosine within a CG sequence
peroxidase activity
DNA replication preinitiation complex
zygote asymmetric cytokinesis in embryo sac
copper ion export
copper-exporting ATPase activity
maintenance of DNA methylation
aleurone grain
peptide-transporting ATPase activity
NADPH oxidase activity
negative regulation of seed germination
integral to plasma membrane
regulation of gene expression by genetic imprinting
drug transmembrane transport
isoflavone 2'-hydroxylase activity
response to ATP
detoxification of copper ion
positive regulation of translation
chloroplast RNA processing
jasmonate O-methyltransferase activity
oligopeptide transport
negative regulation of flower development
fruit ripening
water transport
DNA mediated transformation
gibberellin metabolic process
carbohydrate transport
xenobiotic-transporting ATPase activity
sugar transmembrane transporter activity
leaf senescence
cellulase activity
fruit dehiscence
ammonium transmembrane transporter activity
ammonium transmembrane transport
GTPase activity
nutrient reservoir activity
regulation of cell proliferation
response to oxidative stress
methionine metabolic process
plant-type vacuole
indole glucosinolate metabolic process
polygalacturonase activity
aromatase activity
peroxisome
gibberellin 20-oxidase activity
isopenicillin-N synthase activity
metallocarboxypeptidase activity
anther dehiscence
ATP catabolic process
triglyceride lipase activity

7C

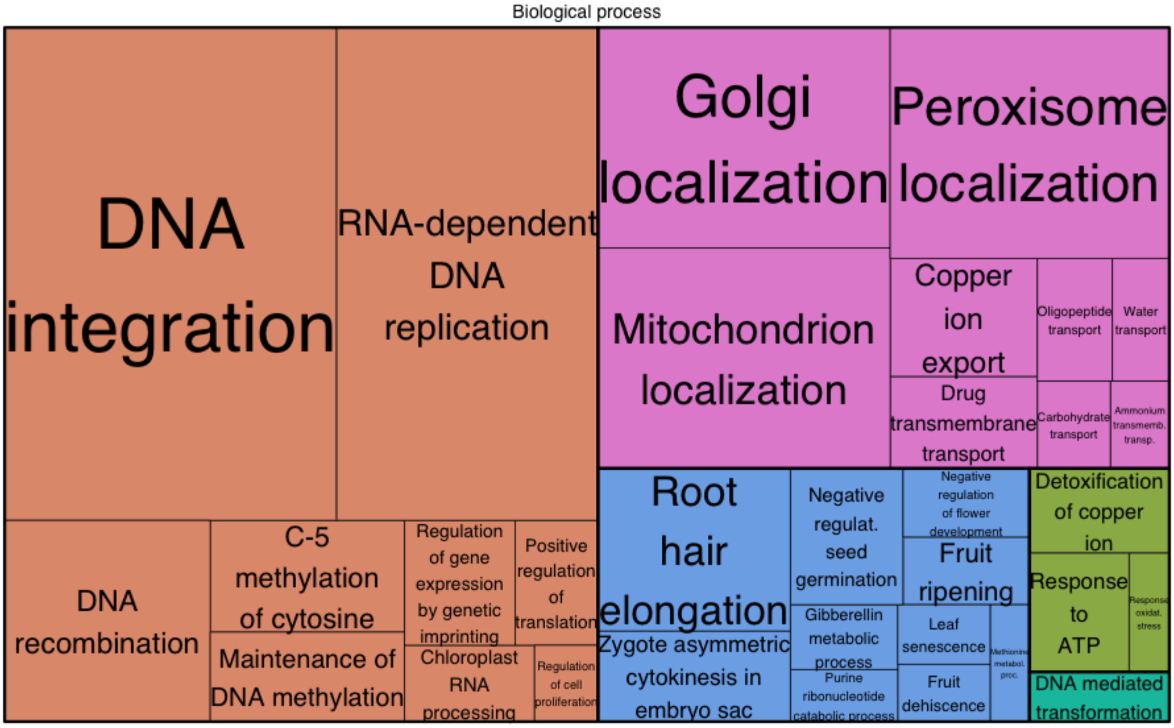
Biological process
DNA integration
RNA-dependent DNA replication
DNA recombination
C-5 methylation of cytosine
Maintenance of DNA methylation
Regulation of gene expression by genetic imprinting
Positive regulation of translation
Chloroplast RNA processing
Regulation of cell proliferation
Golgi localization
Peroxisome localization
Mitochondrion localization
Copper ion export
Oligopeptide transport
Water transport
Drug transmembrane transport
Carbohydrate transport
Ammonium transmemb. transp.
Root hair elongation
Negative regulat. seed germination
Negative regulation of flower development
Fruit ripening
Gibberellin metabolic process
Leaf senescence
Zygote asymmetric cytokinesis in embryo sac
Purine ribonucleotide catabolic process
Fruit dehiscence
Detoxification of copper ion
Response to ATP
DNA mediated transformation

7D

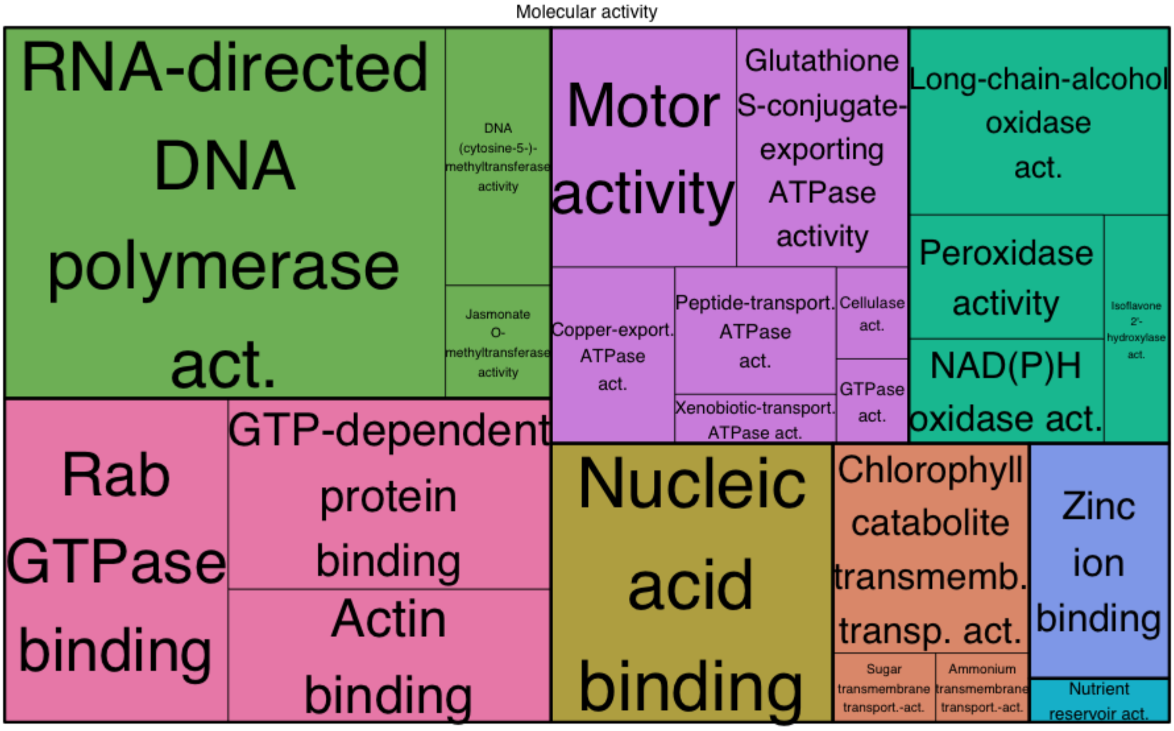
Molecular activity
RNA-directed DNA polymerase act.
DNA (cytosine-5-)-methyltransferase activity
Jasmonate O-methyltransferase activity
Motor activity
Glutathione S-conjugate-exporting ATPase activity
Copper-export. ATPase act.
Peptide-transport. ATPase act.
Cellulase act.
Xenobiotic-transport. ATPase act.
GTPase act.
Long-chain-alcohol oxidase act.
Peroxidase activity
Isoflavone 2'-hydroxylase act.
NAD(P)H oxidase act.
Rab GTPase binding
GTP-dependent protein binding
Actin binding
Nucleic acid binding
Chlorophyll catabolite transmemb. transp. act.
Sugar transmembrane transport.-act.
Ammonium transmembrane transport.-act.
Zinc ion binding
Nutrient reservoir act.

Figure 8. Red clover gene duplication events. Close events are represented together as thicker lines.

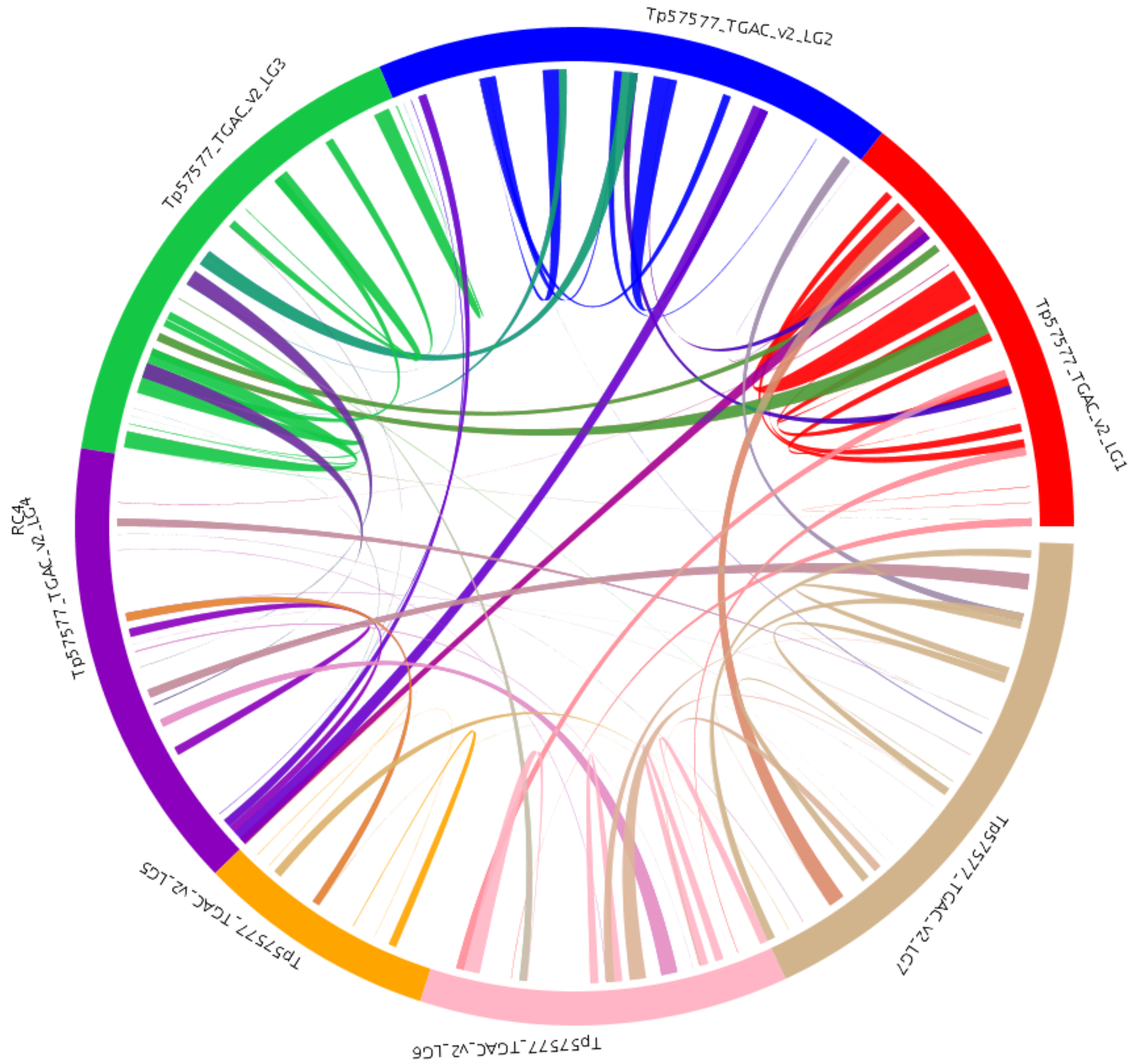

Figure 9. Gene duplication events in red clover. (A) Frequency of Gene duplication events with time (Kimura rates) between chromosomes in the red clover genome. (B) Frequency of Gypsy duplication events with time (Kimura rates) in the red clover and *M. truncatula* genomes.

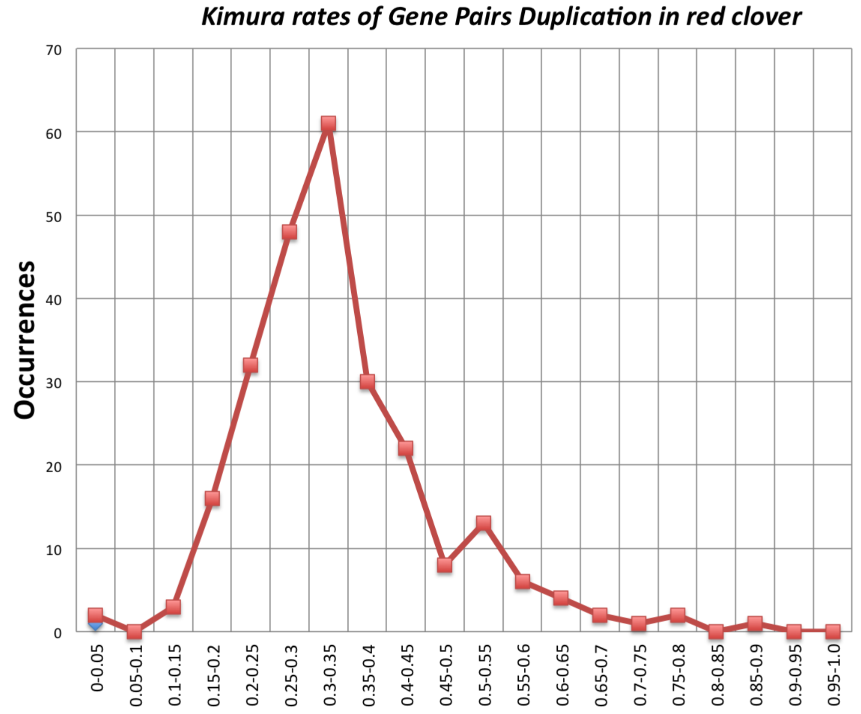

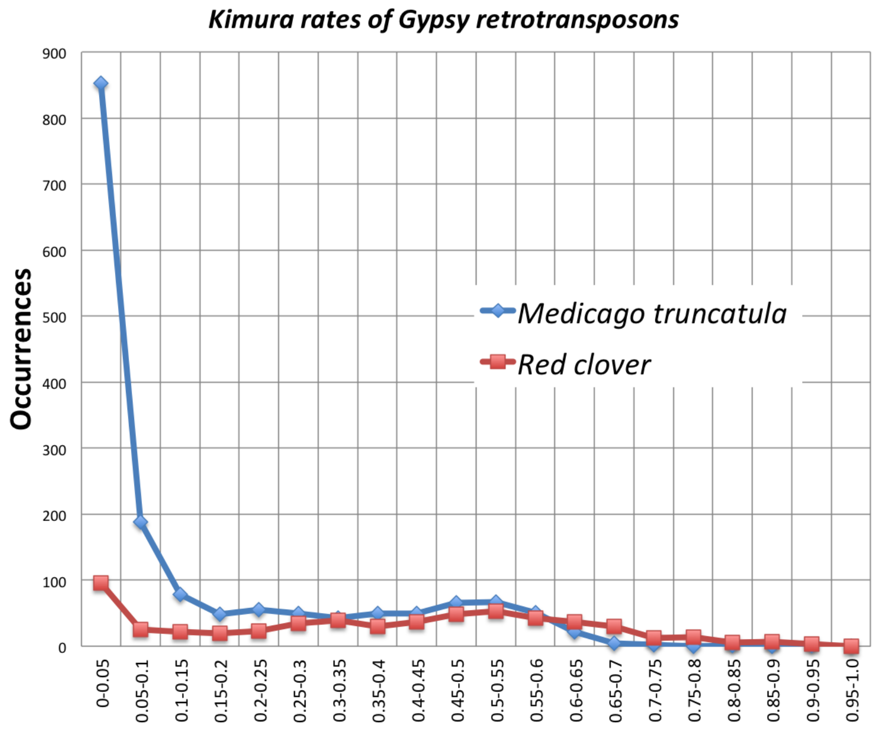

Figure 10. Distribution of the distance from red clover genes to the closest *Gypsy* or *Copia* element, for the whole set of genes (Top panel) or duplicated gene pairs (Bottom panel).

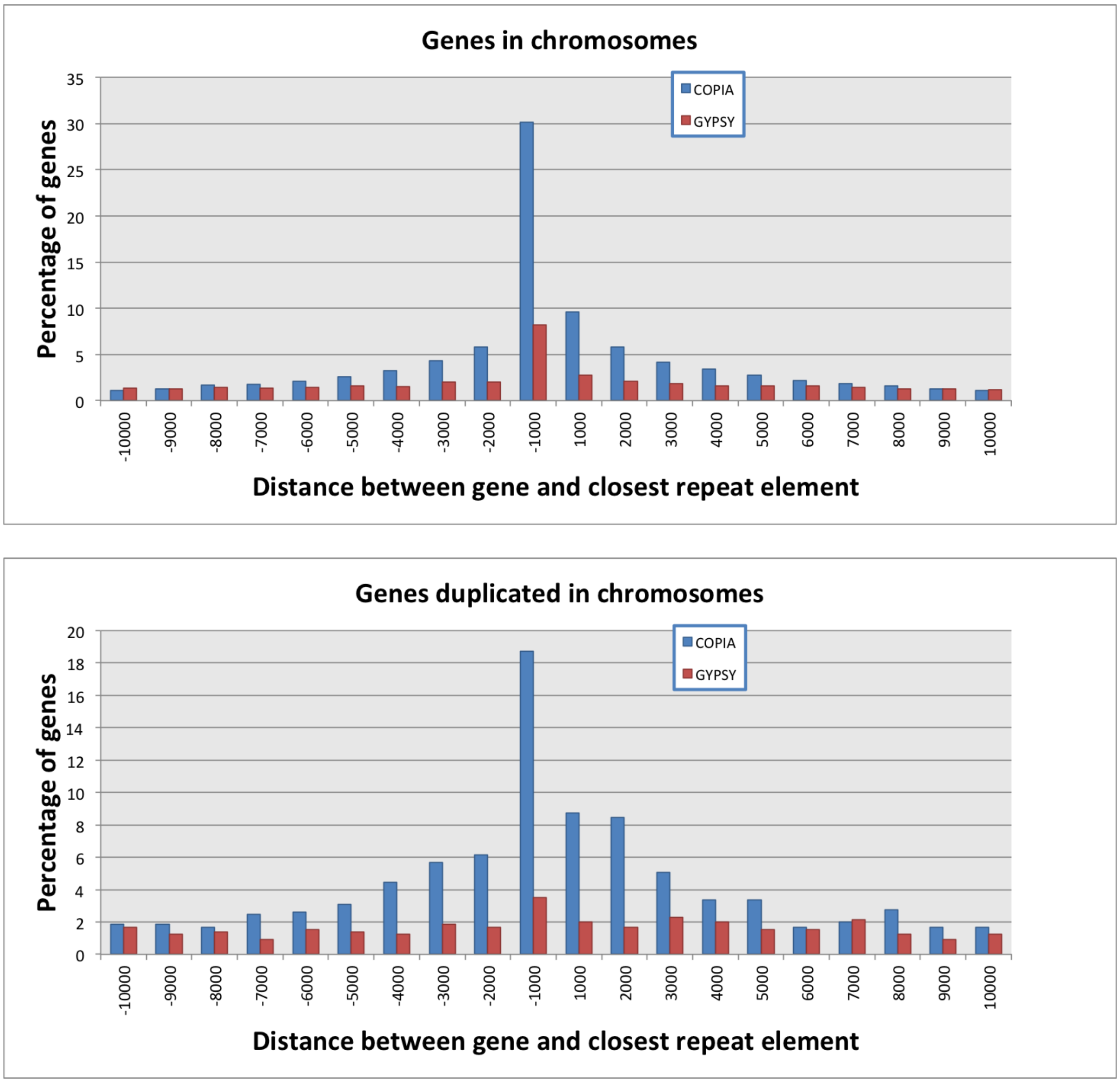

Figure 11. Biosynthesis of formononetin in plants. Schematic representation of the (A) formononetin biosynthesis pathway and (B) formononetin interconversion pathway.

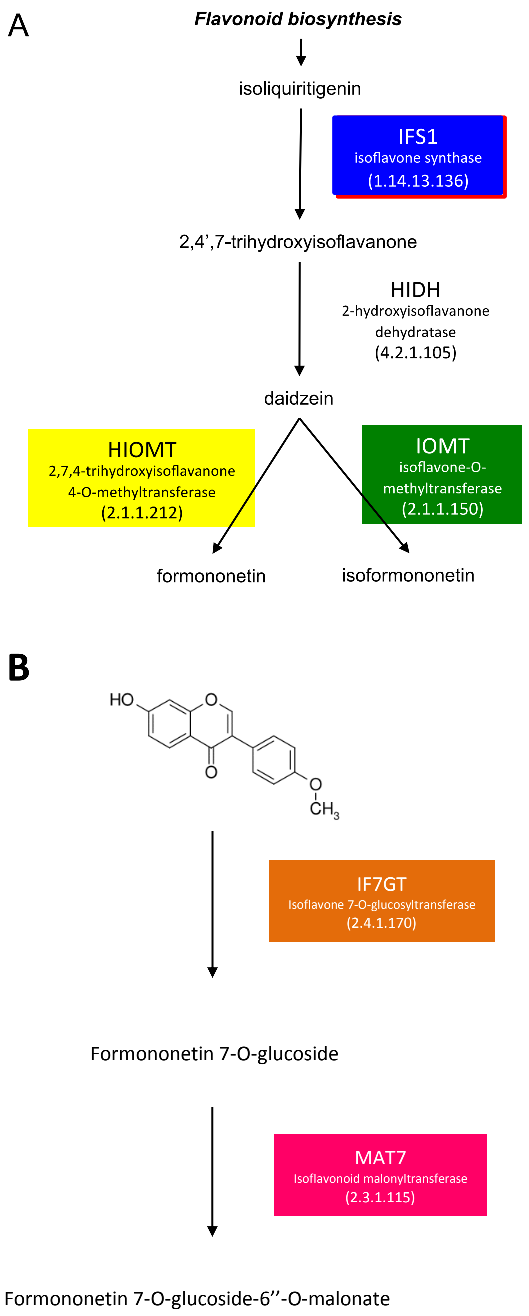

Figure 12. Phylogenetic tree of 2-hydroxyisoflavanone-dehydratase (HIDH) (4.2.1.105) in red clover, soybean (Glyma), common bean (Phvu) and *M. truncatula* (Medtr). The outgroup is represented by *A. thaliana* carboxylesterase.

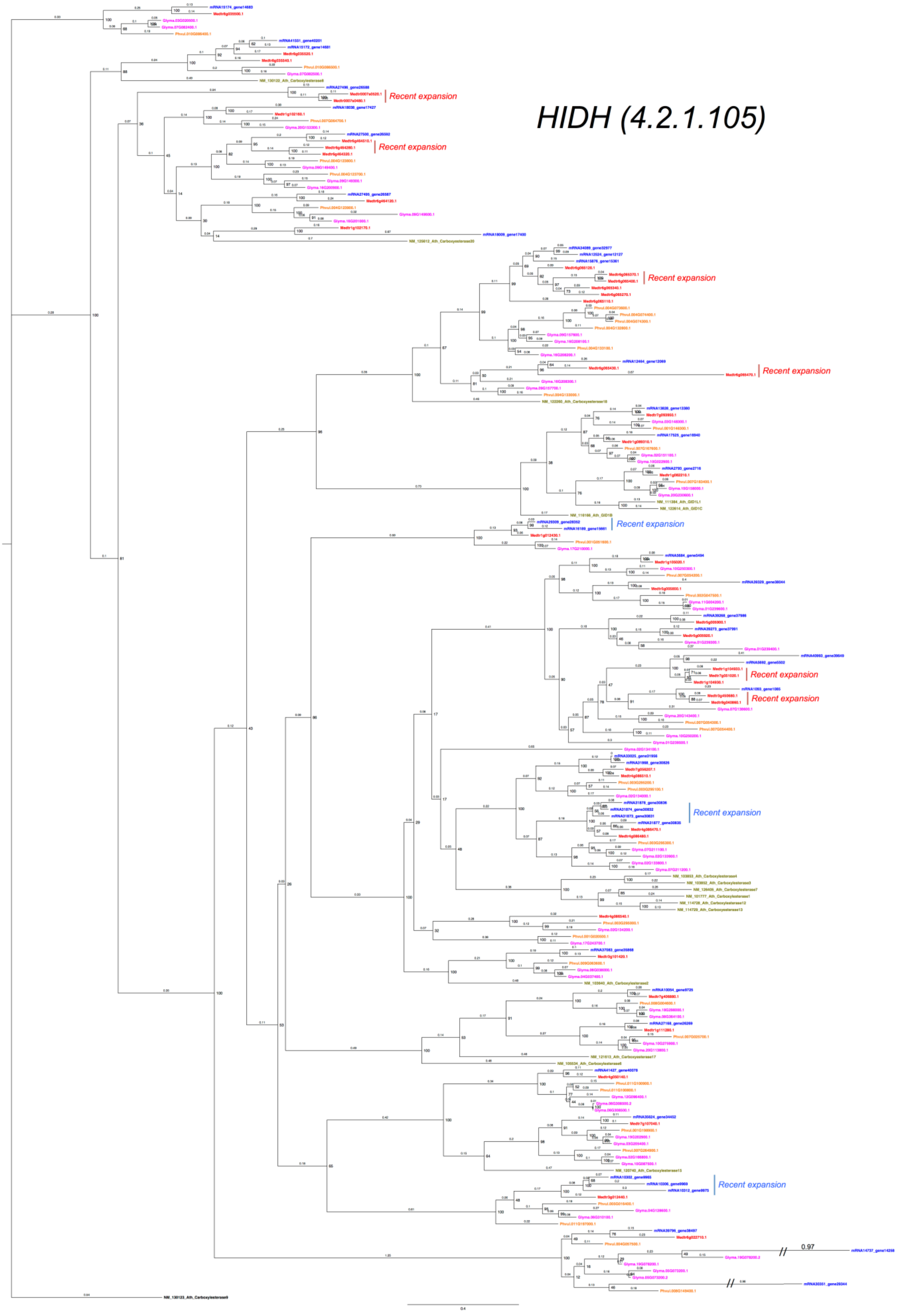

Figure 13. Spatial distribution of four clusters of genes encoding enzymes involved in formononetin biosynthesis in red clover and *M. truncatula*.

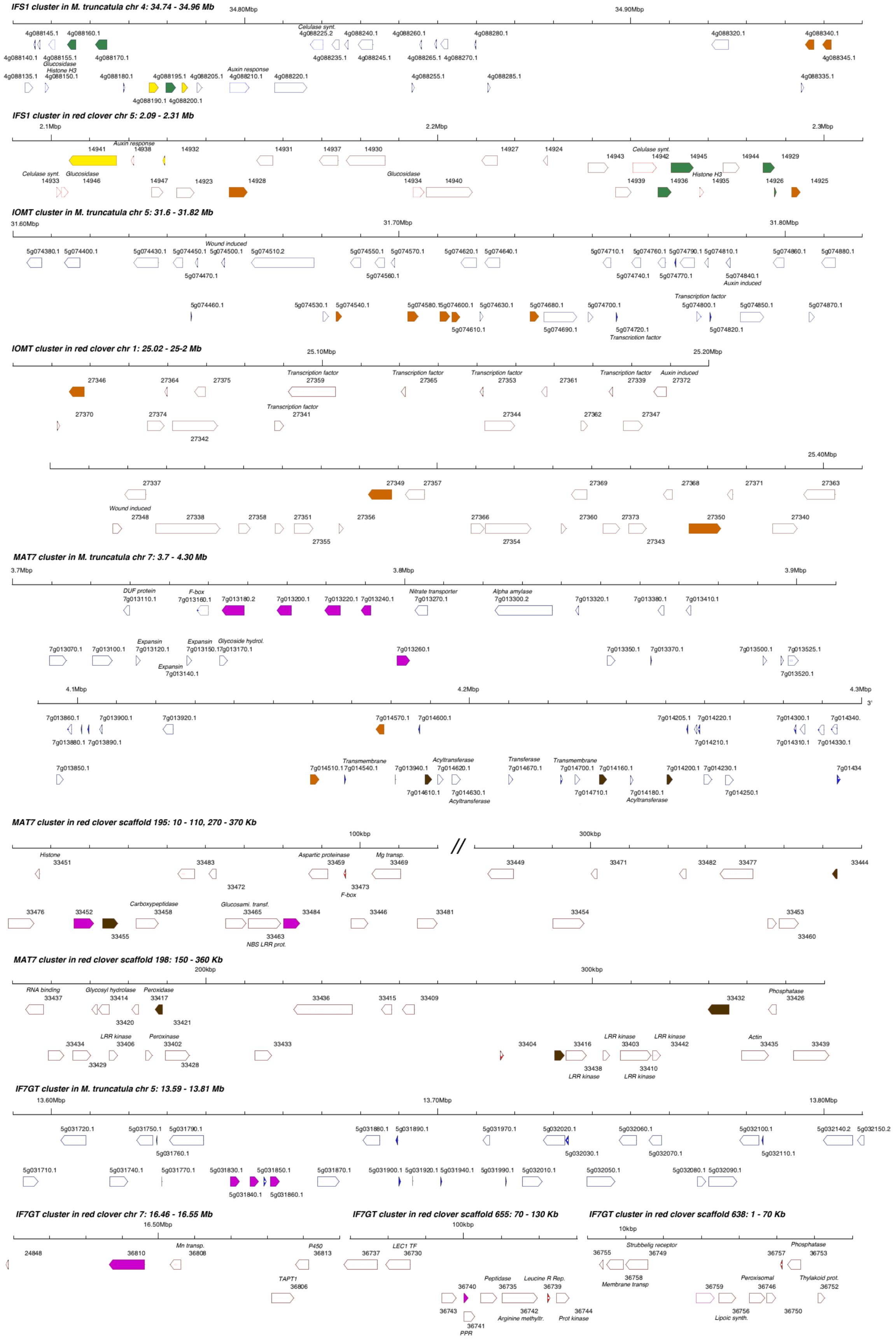

Figure 14. Phylogenetic tree of isoflavone synthase (IFS1) (1.14.13.136) in red clover, soybean (Glyma), common bean (Phvu) and *M. truncatula* (Medtr). The AF532999 gene from *Pisum sativum* provides the outgroup.

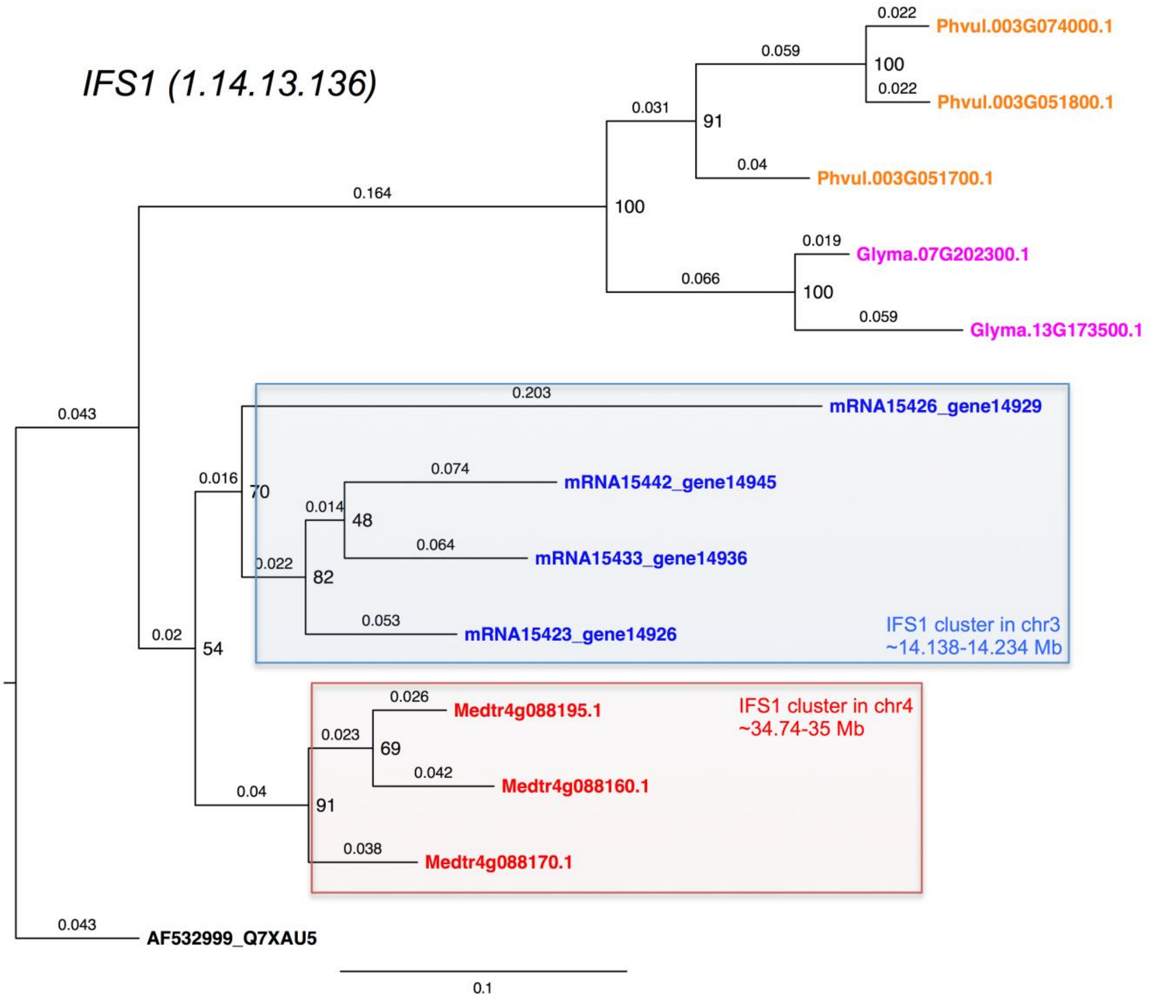

Figure 15. Phylogenetic tree of isoflavone O-methyltransferase (IOMT) (2.1.1.150) in red clover, soybean (Glyma), common bean (Phvu) and *M. truncatula* (Medtr). AT4G35150.1 and AT4G35160.1 are outgroups from *A. thaliana*.

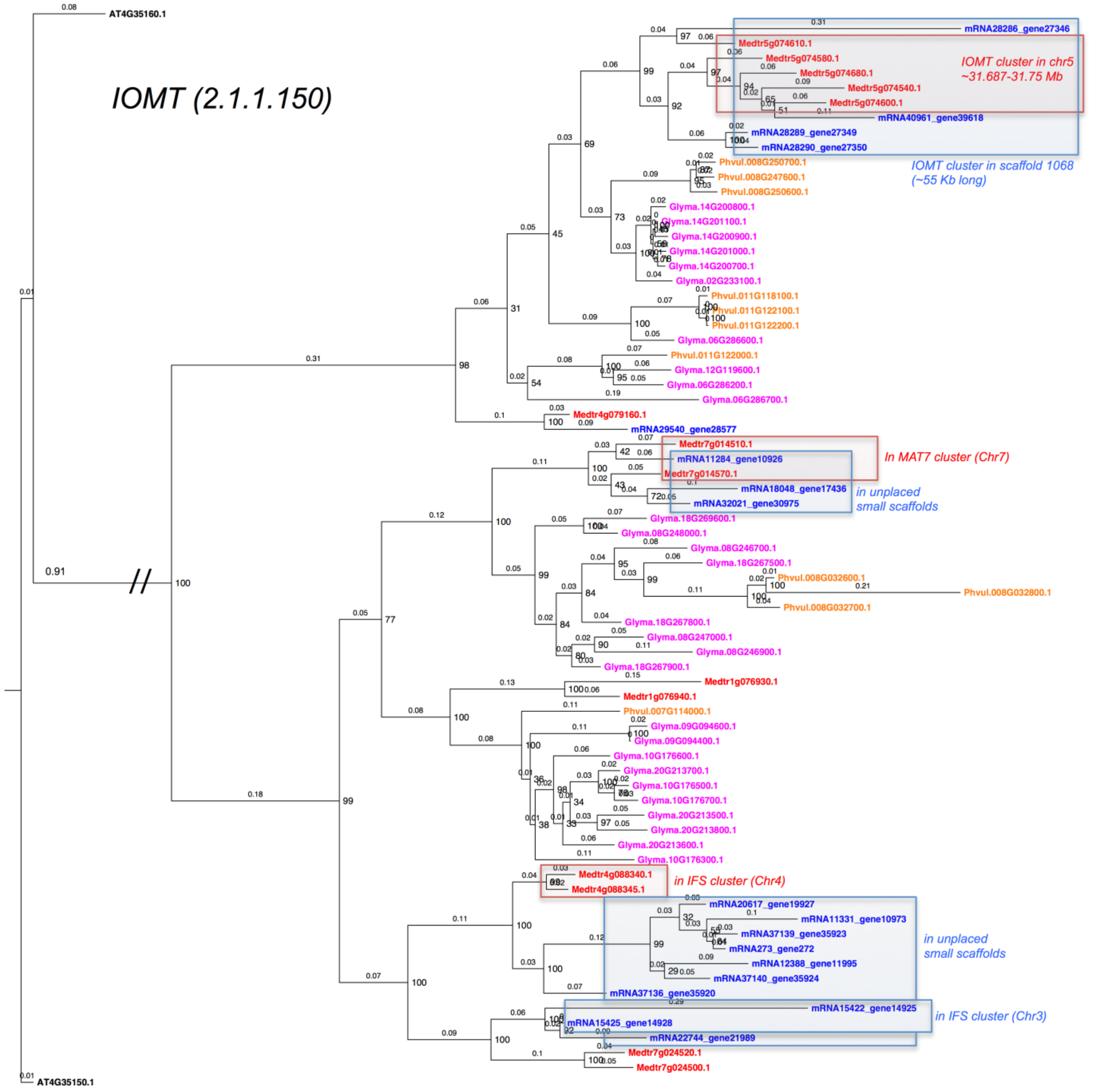

Figure 16. Phylogenetic tree of 2,7,4'-trihydroxyisoflavanone 4'-O-methyltransferase (HIOMT) (2.1.1.212) in red clover, soybean (Glyma), common bean (Phvu) and *M. truncatula* (Medtr). AB091686_mRNA_Q84KK4_extraction from *L. japonicus* is the outgroup.

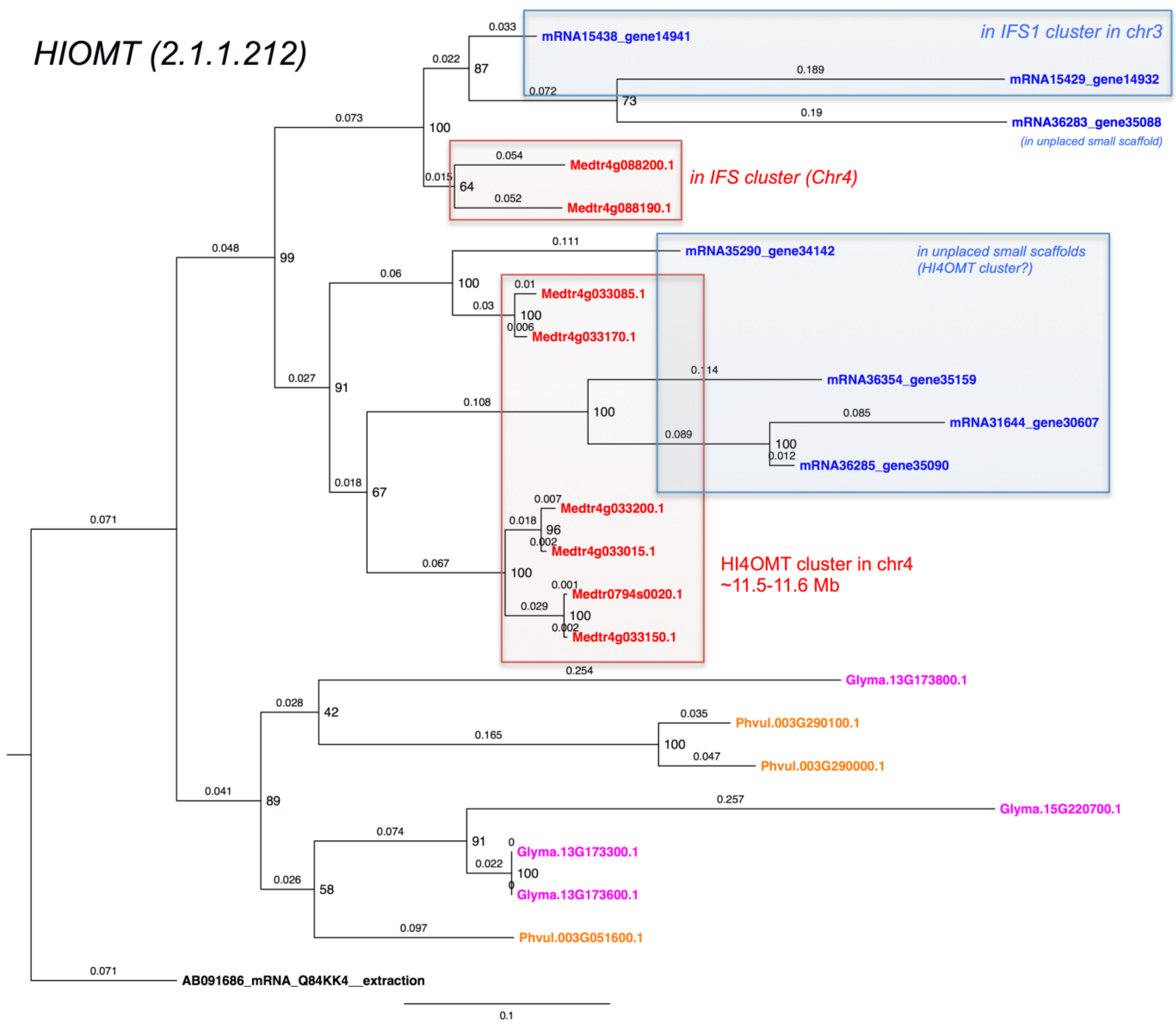

Figure 17. Phylogenetic tree of isoflavone 7-O-glucosyltransferase (IF7GT) (2.4.1.170) in in red clover, soybean (Glyma), common bean (Phvu) and *M. truncatula* (Medtr). The outgroup is AT1G1040 from *A. thaliana*.

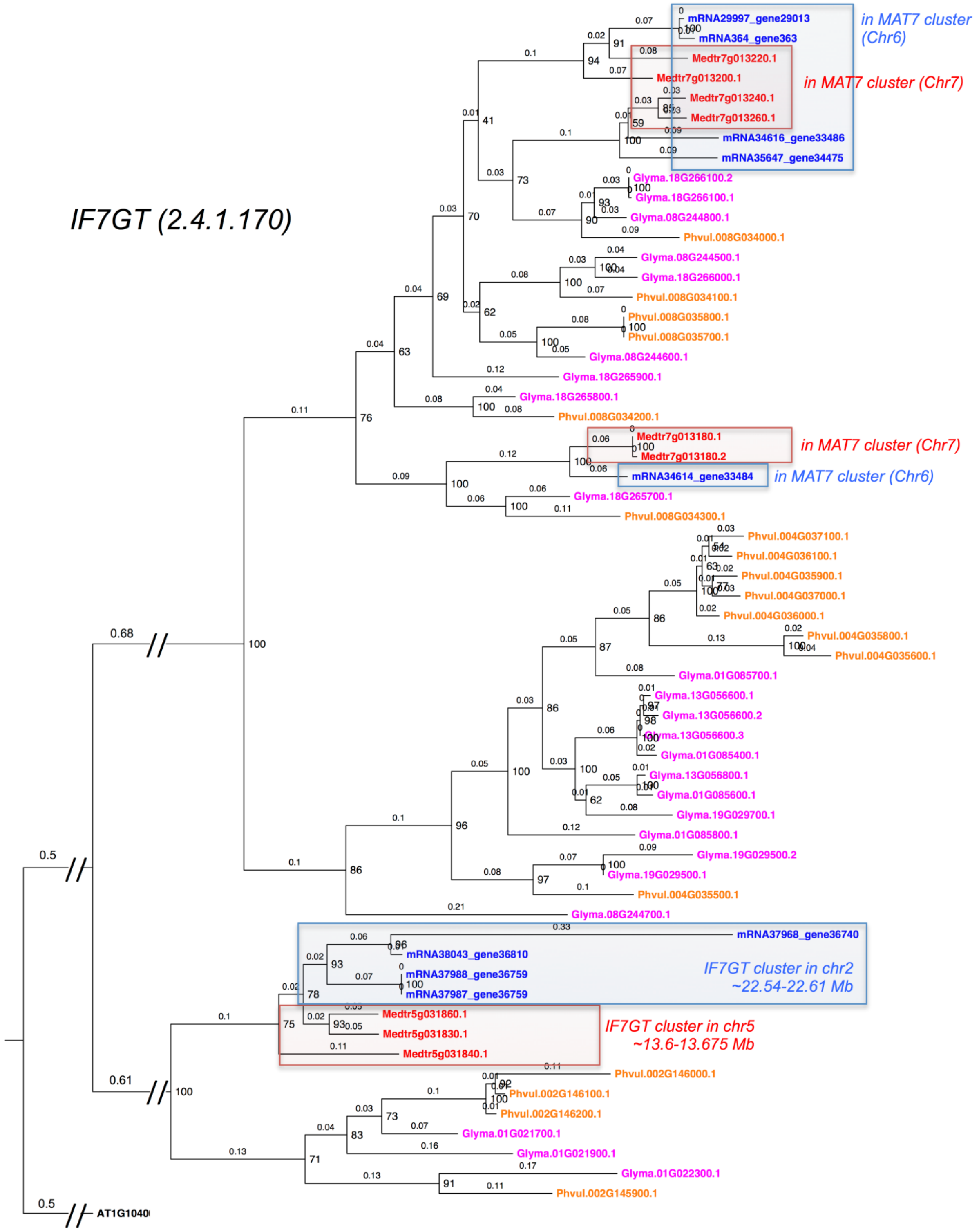

Figure 18. Phylogenetic tree of Isoflavonoid malonyltransferase (MAT7) (2.3.1.115) in red clover, soybean (Glyma), common bean (Phvu) and *M. truncatula* (Medtr). The *Ricinus communis* gene is the outgroup.

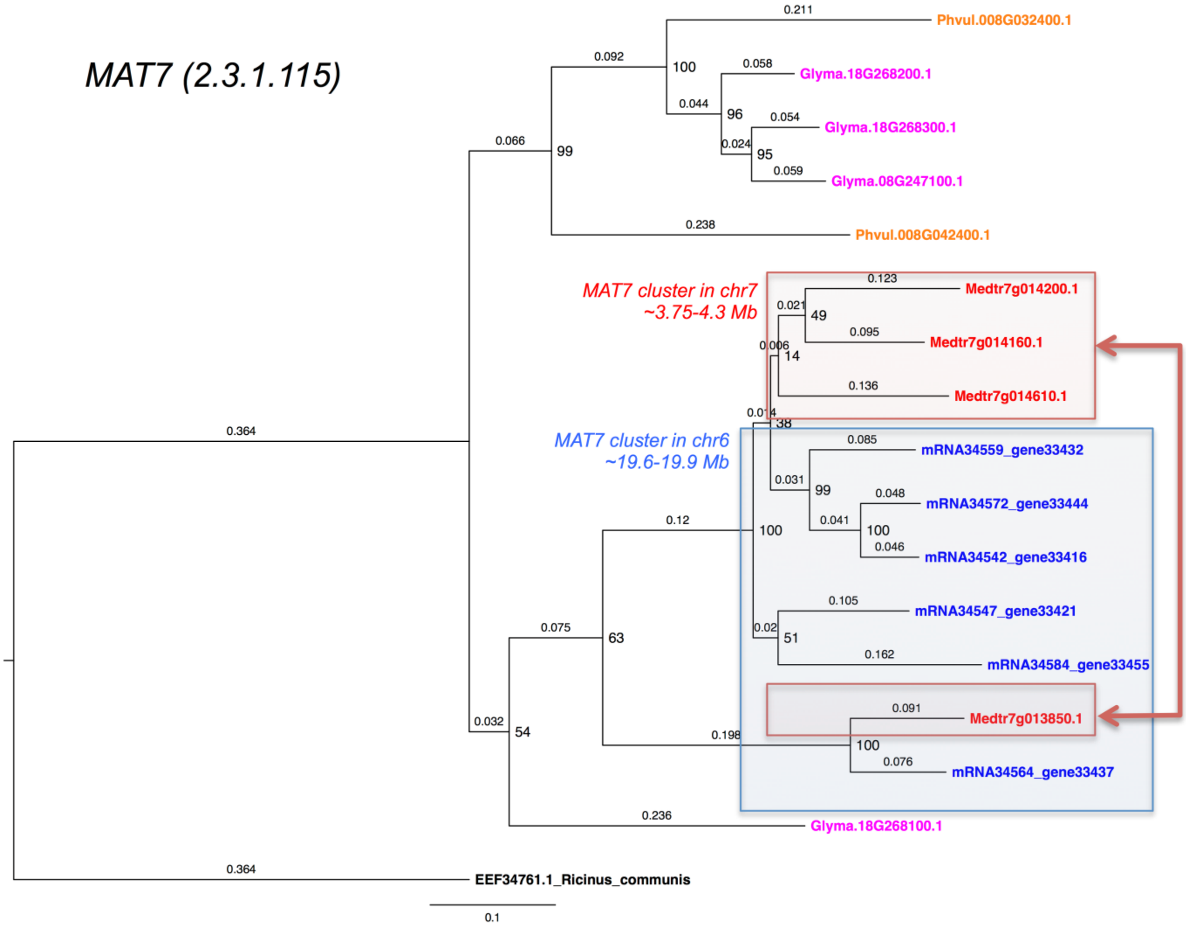

Figure 19. Phylogenetic tree of PPO protein family in legume species. The three transcripts isolated from red clover by Sullivan et al (2004)[2] are in purple, the three extra transcripts proposed by Winters et al (2009)[3] are in orange, and the five genes in the red clover genome are in red. PPO genes identified in *M. truncatula* are indicated in blue. PPO2 genes from *Populus balsamifera* provide outgroups.

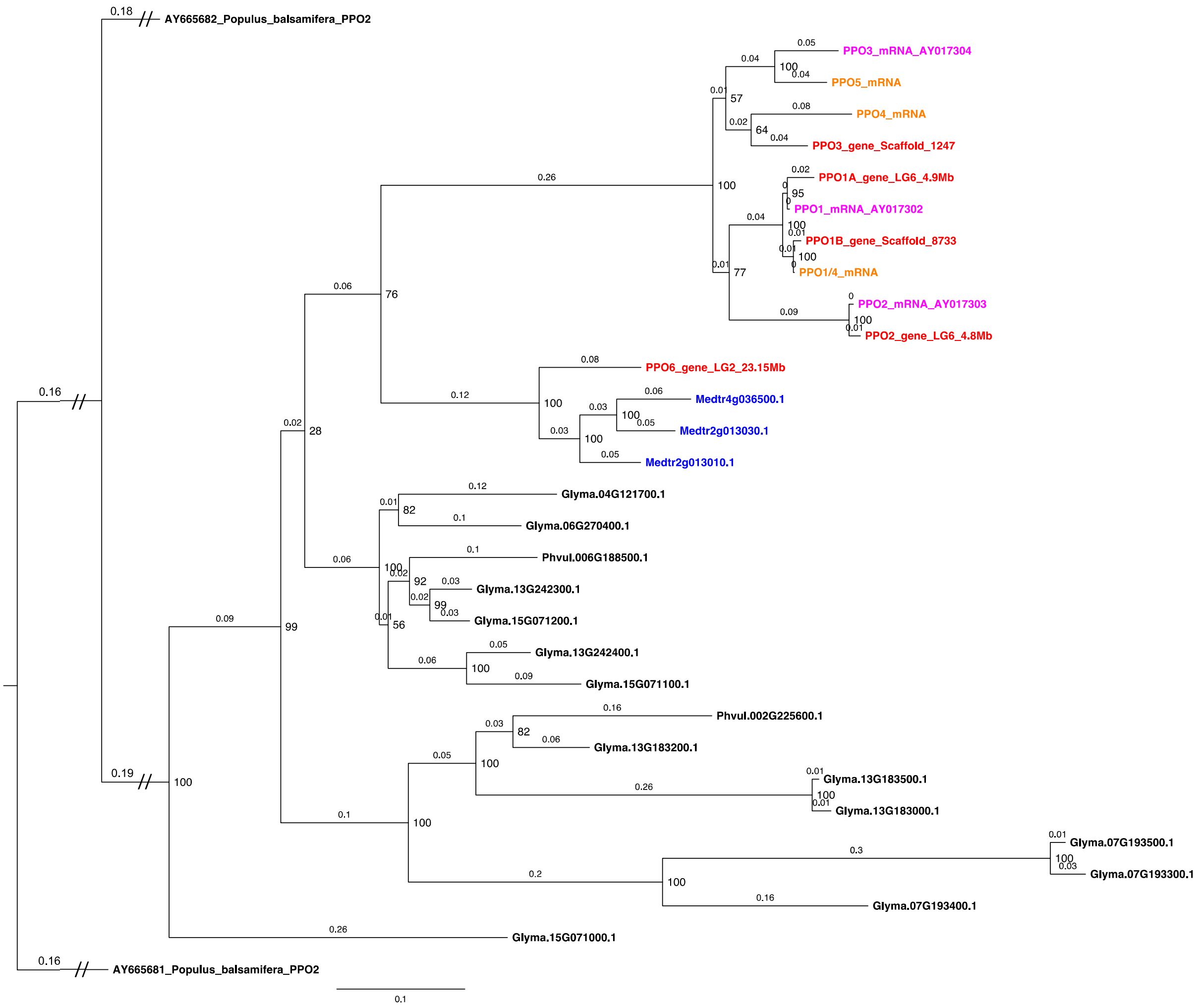

Figure 20. Microsynteny between *M. truncatula* and red clover in the PPO region.

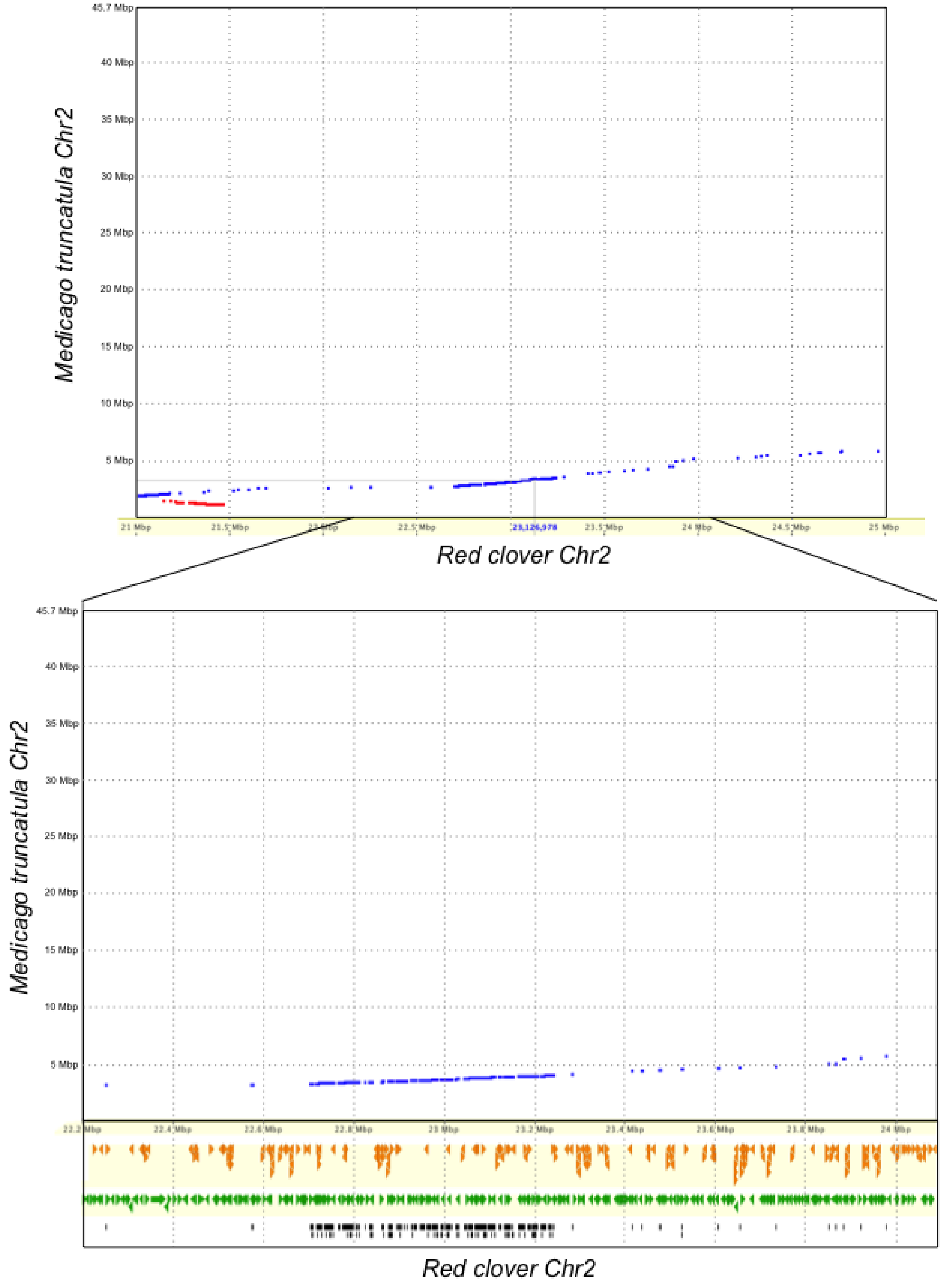

Figure 21. Linkage disequilibrium in a synthetic population of red clover. A: Predicted decay of LD in the seven chromosomes of red clover. B: LD heatmap and landscape plots of the red clover chromosomes. The graphs were generated as described[50].

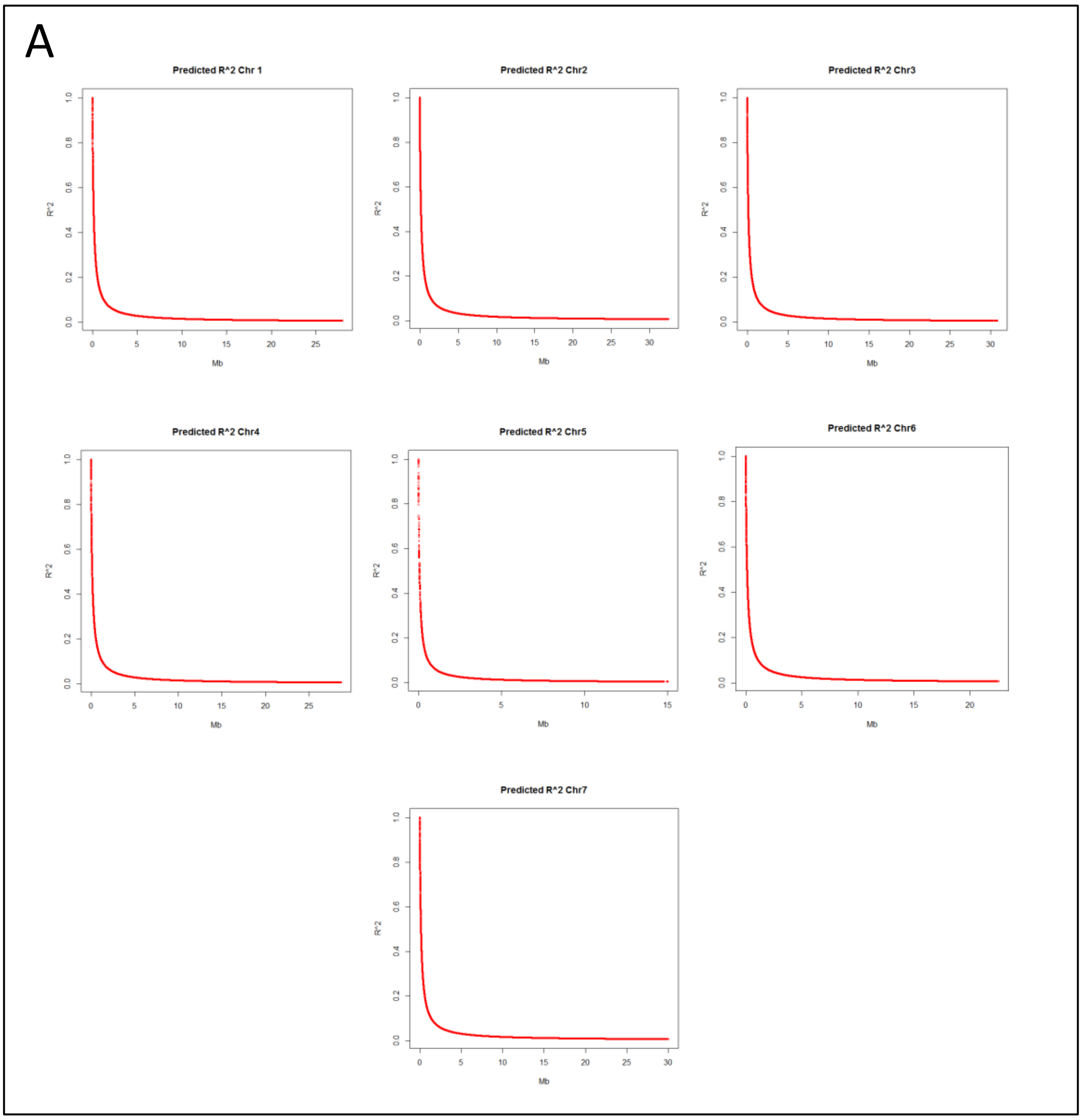

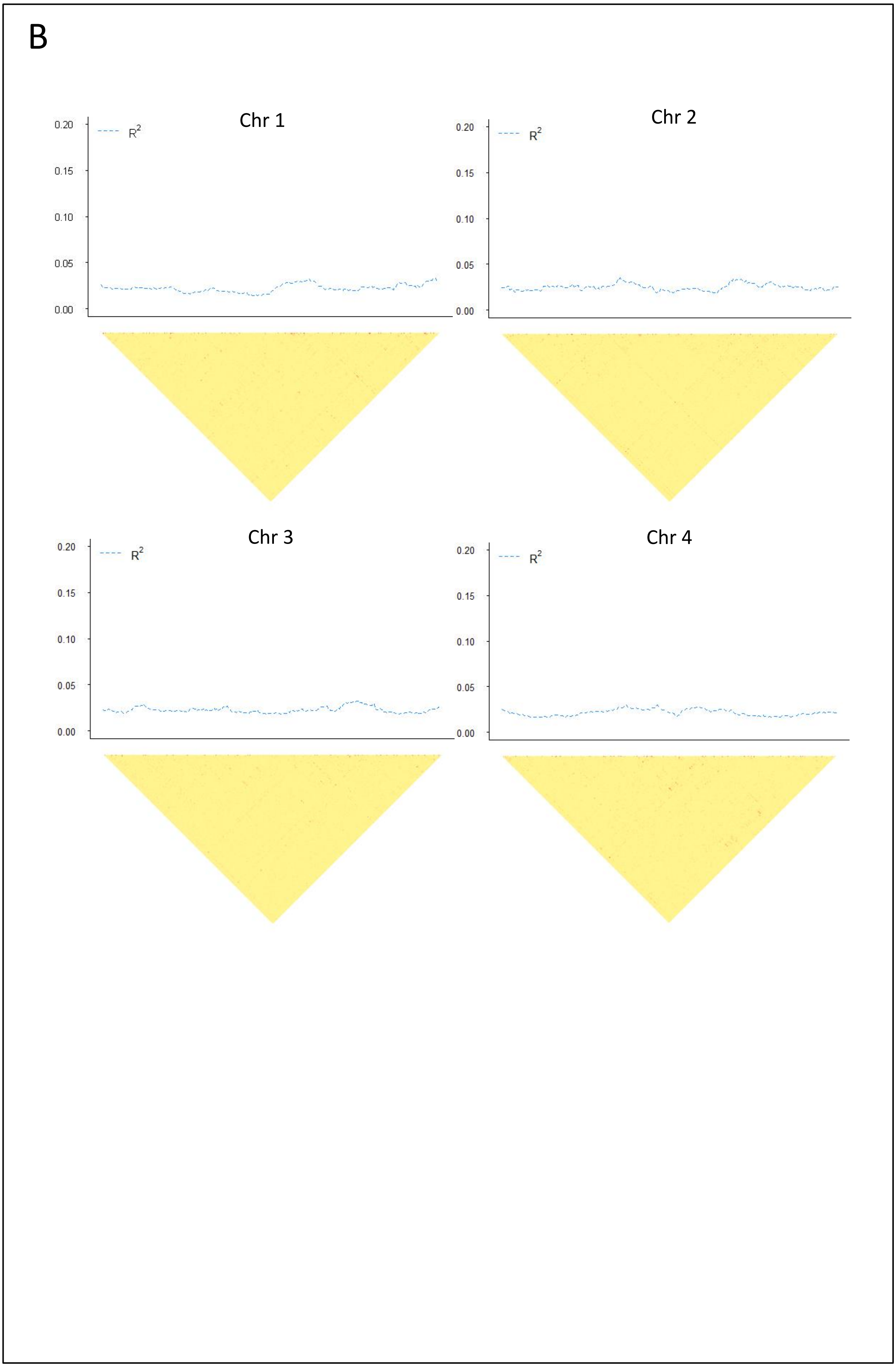

B
Chr 1
Chr 2
Chr 3
Chr 4
R2
0.20
0.15
0.10
0.05
0.00

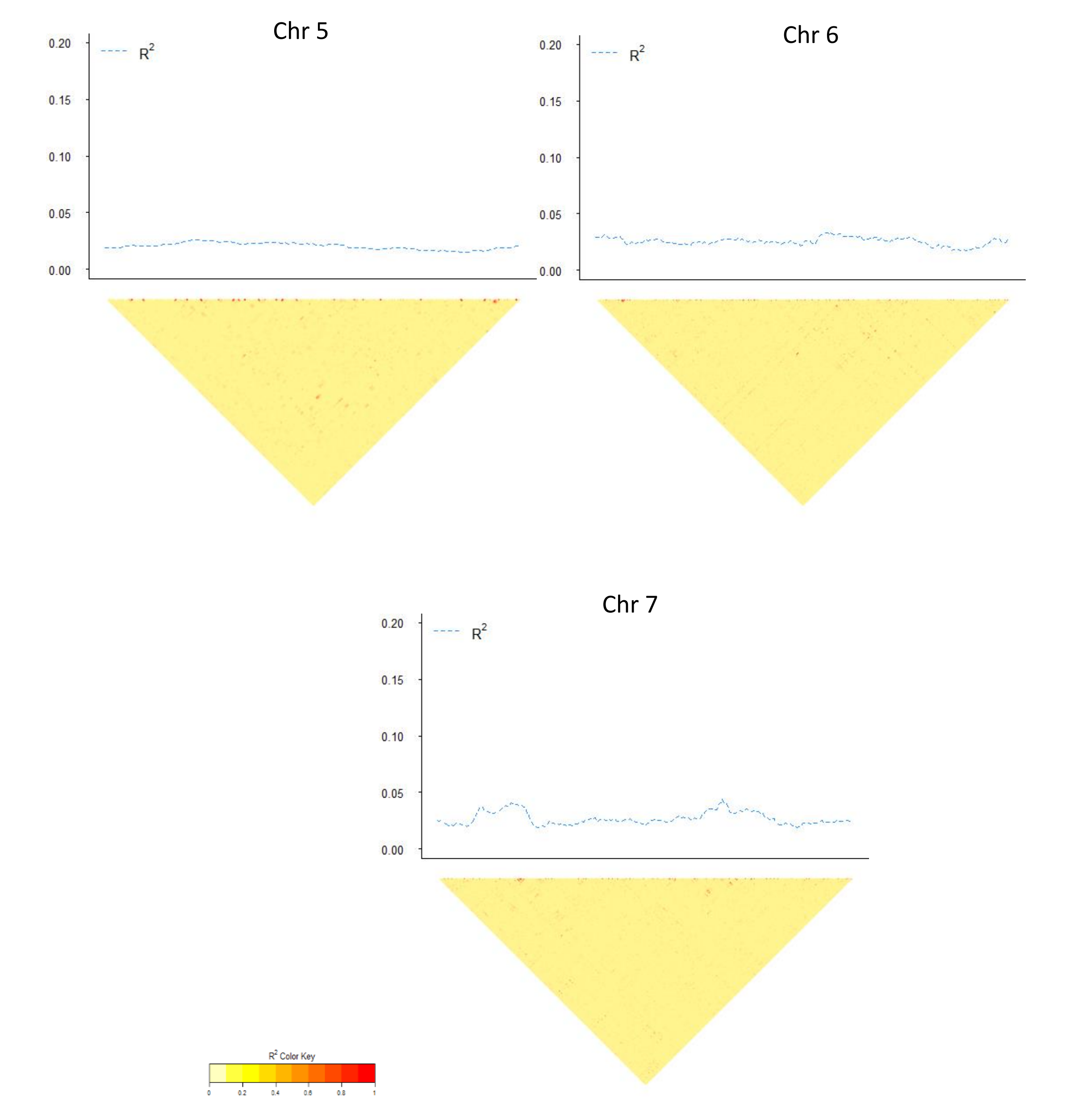
Chr 5
0.20
0.15
0.10
0.05
0.00
R2
Chr 6
0.20
0.15
0.10
0.05
0.00
R2
Chr 7
0.20
0.15
0.10
0.05
0.00
R2
R2 Color Key
0
0.2
0.4
0.6
0.8
1

Figure 22. PCA analysis of red clover synthetic population. The analysis is based on GBS data for the 86 genotypes of the red clover variety "Lea". The two populations were deduced from analysis using the STRUCTURE programme[4].

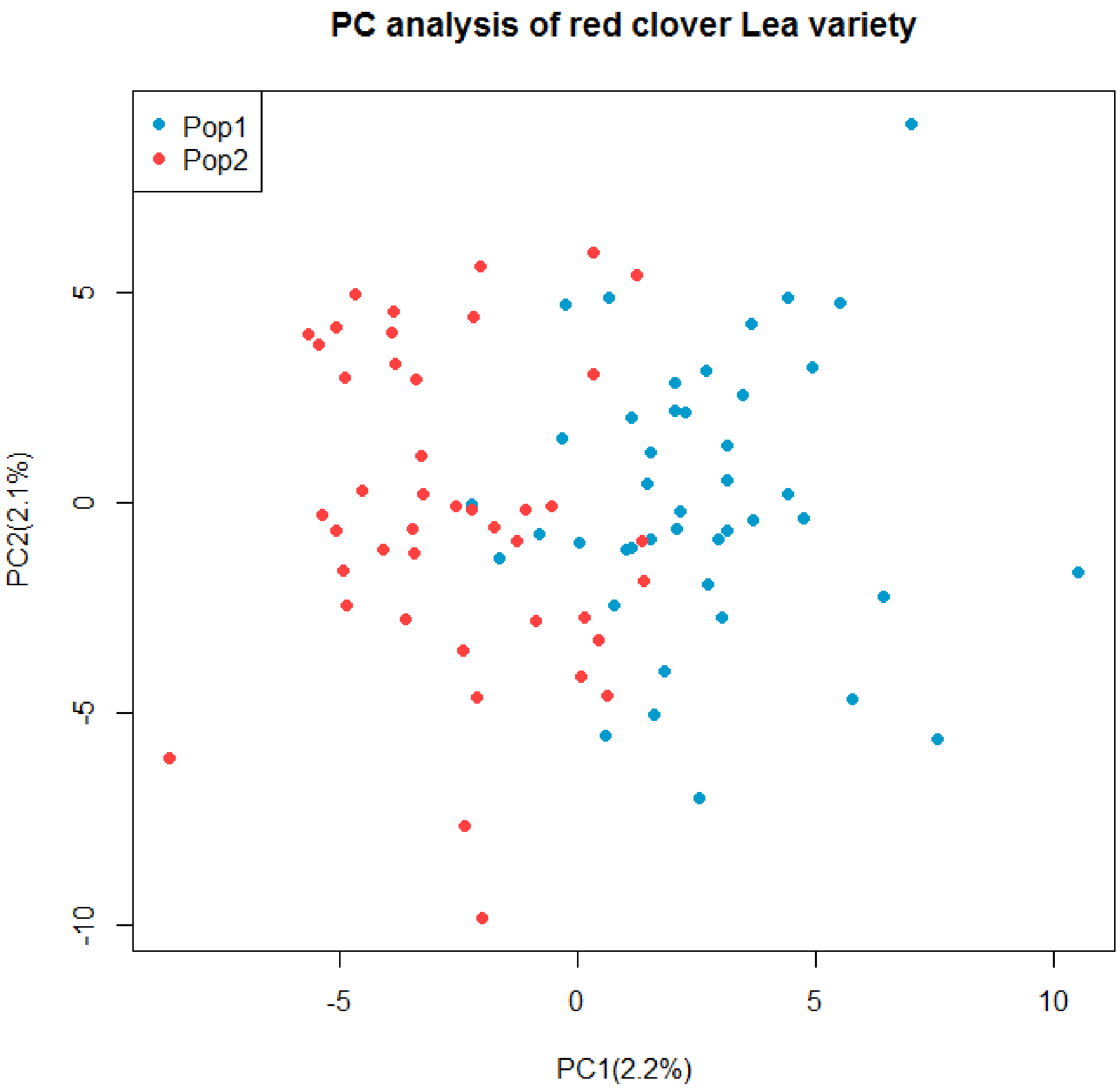

Figure 23. Outline of the annotation pipeline.

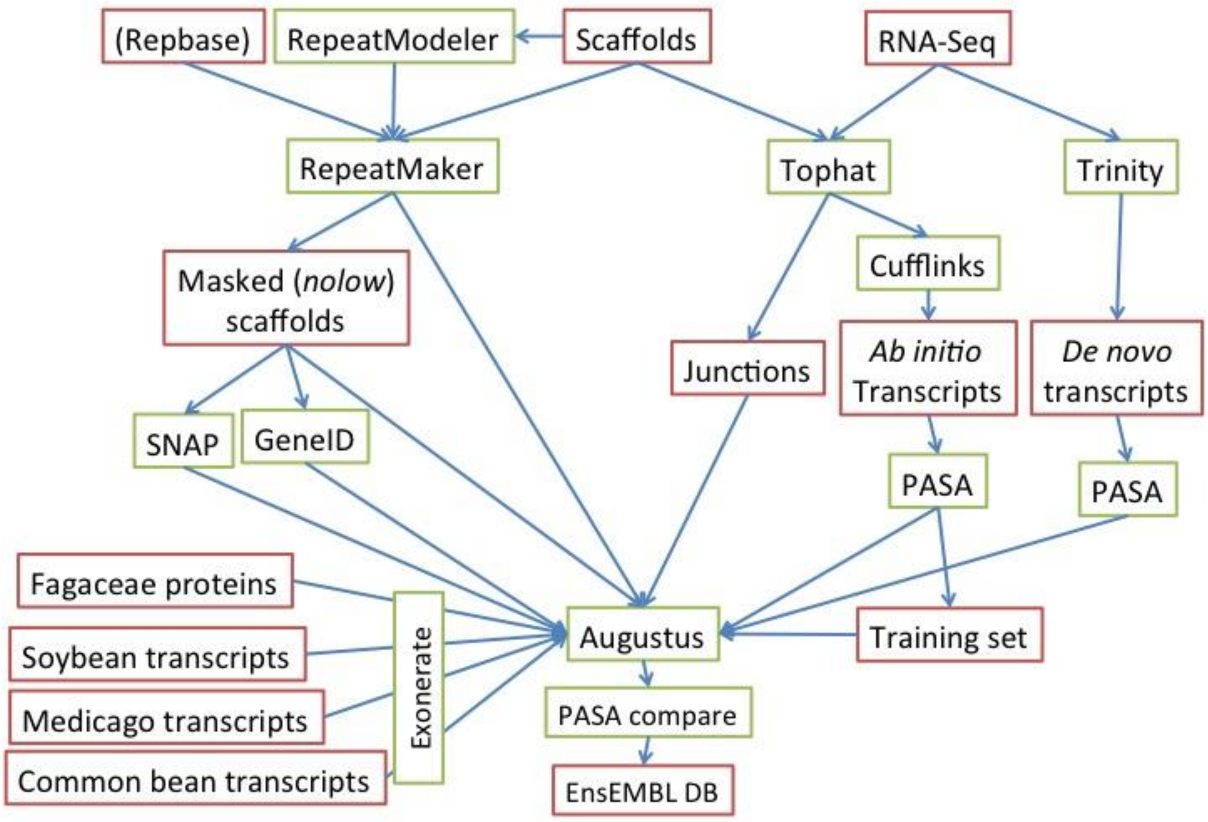

Supplementary Tables

Table 1. Statistics of the WGS, final assembly and pseudo-molecules.

Version	Sequences (Total)	Seqs >N50	N80	N50	N20	Max	Total (Mb)	Placed (Mb)
**WGS (unfiltered)**	347,062	493	1,320	167,200	485,424	1,636,054	362.7	0
**WGS (>500bp)**	39,904	353	25,874	223,063	533,285	1,636,054	309	0
**Final**	39,051	7	29,842	13.02 Mb	26.5 Mb	28.17 Mb	309	164.2
**Pseudo-molecules**	7	4	24.71Mb	25.1 Mb	27 Mb	28.17Mb	164.2	164.2

Table 2. Analysis of the Kmers shared or unique between and within different assembly strategies.

**Assembly**	**Reference: Platanus**	**Alternative: ABySS**
Kmers	349,123,999	522,570,659
... of the previous, are unique/distinct	*...279,627,096 (80.1%)*	*...319,174,661 (61.1%)*
Kmers found in the other assembly	330,745,871 (94,7%)	455,166,458 (87.1%)
Kmers NOT found in the other assembly	18,378,128 (5.3%)	67,404,201 (12.9%)
... of the previous, are unique/distinct	*...18,113,106 (98.6%)*	*...57,660,671 (85.5%)*

Table 3. Position and functional annotation of the genome (MS Excel file).

- Sheet1: Gene/transcripts annotation using Blast2GO, Uniprot homologous, Interpro, eggNOG, and ORF description
- Sheet2: Transcripts correspondence in eggNOG gene clusters, cluster function, cluster family.
- Sheet3: 1,253 transcripts in clusters expanded in red clover in comparison to *M. truncatula*.

Table 4. Classification and proportion of repetitive content in the red clover genome and comparison with other legume genomes.

		Red clover			Medicago truncatula		Phaseolus vulgaris		Glycine max	
	Superfamily	Coverage (bp)	Fraction genome (% of 309 Mbp)	Fraction in pseudo-molecules (% of 164 Mbp)	Coverage (bp)	Fraction genome (% of 389 Mbp)	Coverage (bp)	Fraction genome (% of 472 Mbp)	Coverage (bp)	Fraction genome (% of 955 Mbp)
Class 1 TEs	Gypsy	7566938	2.45	1.65	38954999	10.01	131786780	27.92	269298878	28.19
	Copia	24391421	7.89	5.99	30381006	7.81	79334021	16.81	154704057	16.19
	SINEs	1480120	0.48	0.47	3085624	0.79	164280	0.03	1340657	0.14
	LINEs	19637581	6.35	5.57	25763983	6.62	50090542	10.61	25568379	2.68
	Total Class 1	63552730	20.57	16.29	112990132	29.05	286983344	60.8	479305937	50.19
Class 2 (DNA) TEs	hAT	9500001	3.07	2.99	5920232	1.52	6014080	1.27	17306917	1.81
	Harbinger/PIF	4926009	1.59	1.70	7055779	1.81	3268021	0.69	2389726	0.25
	MULE	11052932	3.58	3.56	29629191	7.62	4795780	1.02	30491071	3.19
	Stowaway	3803945	1.23	1.26	3139504	0.81	0	0.00	494603	0.05
	Pogo	1359180	0.44	0.45	0	0.00	0	0.00	0	0.00
	CMC_EnSpm	1220979	0.40	0.33	1942964	0.50	11950619	2.53	28217241	2.95
	Helitron	4505094	1.46	1.35	5911950	1.52	1590726	0.34	3162793	0.33
	Other	22539391	7.29	7.02	19516885	5.02	22539087	4.78	57559831	6.03
	Total Class 2	58907531	19.06	18.65	73116505	18.80	50158313	10.63	139622182	14.62
UnclassifTE	Unclassif TE	5116317	1.66	1.56	2222114	0.57	2465270	0.52	3676862	0.38
Non TEs	Simple reps	1516187	0.49	0.46	1056782	0.27	657868	0.14	1652138	0.17
	Satellites	110986	0.036	0.03	36149	0.01	1126	0.00	764888	0.08
	TOTAL	129226537	41.82	37.01	189498274	48.71	340265921	72.09	626039060	65.55

Table 5. Average pairwise linkage disequilibrium (r^2) in the seven red clover chromosomes at three distances.

Chromosome	0.076 Mb	0.1 Mb	0.5 Mb
**1**	0.31	0.25	0.06
**2**	0.21	0.17	0.04
**3**	0.19	0.15	0.03
**4**	0.24	0.20	0.05
**5**	0.21	0.17	0.04
**6**	0.25	0.20	0.05
**7**	0.22	0.18	0.04

Table 6. Shotgun short-read libraries used in the WGS.

Library	Reads	EBI-ENA Accession
**PE (100 bp)**	101.4e6	ERX946106
**PE (150 bp)**	306.3e6	ERX946107
**SE (150 bp)**	254.6e6	ERX946108, ERX946109
**Overlap PE (150 bp)**	25.6e6	ERX946110
**3 Kb MP (100 bp)**	224.8e6	ERX946085, ERX946084
**5 Kb MP (65 bp)**	4.2e6	ERX946083
**7 Kb MP (100 bp)**	20.4e6	ERX946086
**7 Kb MP (150 bp)**	88.0e6	ERX946087

All shotgun reads and the assembly are deposited in the European Nucleotide Archive (accession PRJEB9186). The genome assembly and annotation can also be downloaded as files (http://dx.doi.org/10.5281/zenodo.17232) or browsed online (http://tgac-browser.tgac.ac.uk/trifolium_pratense).

Supplementary References

1 Ištvánek, J., Jaroš, M., Křenek, A. & Řepková, J. Genome assembly and annotation for red clover (Trifolium pratense; Fabaceae). *American Journal of Botany* **101**, 327-337 (2014).

2 Sullivan, M. L., Hatfield, R. D., Thoma, S. L. & Samac, D. A. Cloning and Characterization of Red Clover Polyphenol Oxidase cDNAs and Expression of Active Protein in Escherichia coli and Transgenic Alfalfa. *Plant Physiology* **136**, 3234-3244 (2004).

3 Winters, A. *et al.* Identification of an extensive gene cluster among a family of PPOs in Trifolium pratense L. (red clover) using a large insert BAC library. *BMC Plant Biology* **9**, 94 (2009).

4 Pritchard, J. K., Stephens, M. & Donnelly, P. Inference of Population Structure Using Multilocus Genotype Data. *Genetics* **155**, 945-959 (2000).
